# Runs of homozygosity analysis of South African sheep breeds from various production systems investigated using OvineSNP50k data

**DOI:** 10.1186/s12864-020-07314-2

**Published:** 2021-01-06

**Authors:** E. F. Dzomba, M. Chimonyo, R. Pierneef, F. C. Muchadeyi

**Affiliations:** 1 Discipline of Genetics, School of Life Sciences, University of KwaZulu-Natal, Private Bag X01, Scottsville, 3209 South Africa; 2 Discipline of Animal & Poultry Science; School of Agricultural, Earth & Environmental Sciences, University of KwaZulu-Natal, Private Bag X01, Scottsville, 3209 South Africa; 3 Agricultural Research Council, Biotechnology Platform, Private Bag X5, Onderstepoort, 0110 South Africa

**Keywords:** Sheep, Production system, SNP genotypes, Runs of Homozygosity, Autozygosity, ROH island

## Abstract

**Background:**

Population history, production system and within-breed selection pressure impacts the genome architecture resulting in reduced genetic diversity and increased frequency of runs of homozygosity islands. This study tested the hypothesis that production systems geared towards specific traits of importance or natural or artificial selection pressures influenced the occurrence and distribution of runs of homozygosity (ROH) in the South African sheep population. The Illumina OvineSNP50 BeadChip was used to genotype 400 sheep belonging to 13 breeds from South Africa representing mutton, pelt and mutton and wool dual-purpose breeds, including indigenous non-descript breeds that are reared by smallholder farmers. To get more insight into the autozygosity and distribution of ROH islands of South African breeds relative to global populations, 623 genotypes of sheep from worldwide populations were included in the analysis. Runs of homozygosity were computed at cut-offs of 1–6 Mb, 6–12 Mb, 12–24 Mb, 24–48 Mb and > 48 Mb, using the R package detectRUNS. The Golden Helix SVS program was used to investigate the ROH islands.

**Results:**

A total of 121,399 ROH with mean number of ROH per animal per breed ranging from 800 (African White Dorper) to 15,097 (Australian Poll Dorset) were obtained. Analysis of the distribution of ROH according to their size showed that, for all breeds, the majority of the detected ROH were in the short (1–6 Mb) category (88.2%). Most animals had no ROH > 48 Mb. Of the South African breeds, the Nguni and the Blackhead Persian displayed high ROH based inbreeding (F_ROH_) of 0.31 ± 0.05 and 0.31 ± 0.04, respectively. Highest incidence of common runs per SNP across breeds was observed on chromosome 10 with over 250 incidences of common ROHs. Mean proportion of SNPs per breed per ROH island ranged from 0.02 ± 0.15 (island ROH224 on chromosome 23) to 0.13 ± 0.29 (island ROH175 on chromosome 15). Seventeen (17) of the islands had SNPs observed in single populations (unique ROH islands). The MacArthur Merino (MCM) population had five unique ROH islands followed by Blackhead Persian and Nguni with three each whilst the South African Mutton Merino, SA Merino, White Vital Swakara, Karakul, Dorset Horn and Chinese Merino each had one unique ROH island. Genes within ROH islands were associated with predominantly metabolic and immune response traits and predomestic selection for traits such as presence or absence of horns.

**Conclusions:**

Overall, the frequency and patterns of distribution of ROH observed in this study corresponds to the breed history and implied selection pressures exposed to the sheep populations under study.

**Supplementary Information:**

The online version contains supplementary material available at 10.1186/s12864-020-07314-2.

## Background

The genetic diversity of South African sheep populations is considered complex having been shaped by multifaceted production systems [1, 2] resulting from a combination of indigenous, commercial and synthetic/composite breeds raised to suit, various and often, extreme production conditions where natural selection forces are at play. Coupled with this have been farmer driven initiatives to crossbreed as an effort to develop breeds that are better suited to produce optimally under the harsh production conditions of the country. Whilst South African sheep genetic resources have been imported and introduced in other countries globally, there has also been movement of breeds into South Africa [3]. The country has a combination of both large- and small-framed breeds where both inbreeding and outbreeding are considered dominant forces moulding their phenotypic appearance. Both natural and artificial selection of sheep, as well as regional variations due to drift, have resulted in sheep breeds that differ extensively in phenotypes.

Production system and within-breed selection pressure have pronounced effects on the genome architecture and may cause reduced genetic diversity and frequency of runs of homozygosity islands [4]. Runs of homozygosity (ROH) are contiguous segments of homozygous genotypes that are present in an individual due to parents transmitting identical haplotypes to their offspring [5]. The extent and frequency of ROHs are useful in providing information about the ancestry of an individual and its population [5, 6] with longer ROHs associated with more recent inbreeding within a pedigree while short ROHs are associated with ancient common ancestors [7, 8]. Shorter ROH can also be used to infer ancient relationships, information which in livestock is often missing due to limited recording. Long runs of homozygosity have been observed to be persistent in inbred individuals, suggestive of unusually low mutation rates, high linkage disequilibrium (LD), and low recombination rates at certain genomic regions [9]. ROH accumulation in certain genomic positions has been used to analyze the demographic history in humans [10, 11] and livestock populations [12, 13]. A study also used ROH to compare and characterize beef and dairy cattle breeds [14]. ROHs are also common in regions under positive selection and as such studies have associated accumulation of ROHs at specific loci to directional selection [13, 15]. In a number of studies, ROH have been used to estimate inbreeding levels and infer on signatures of selection and genetic adaptation to production conditions [16–18].

**Fig. 1:**
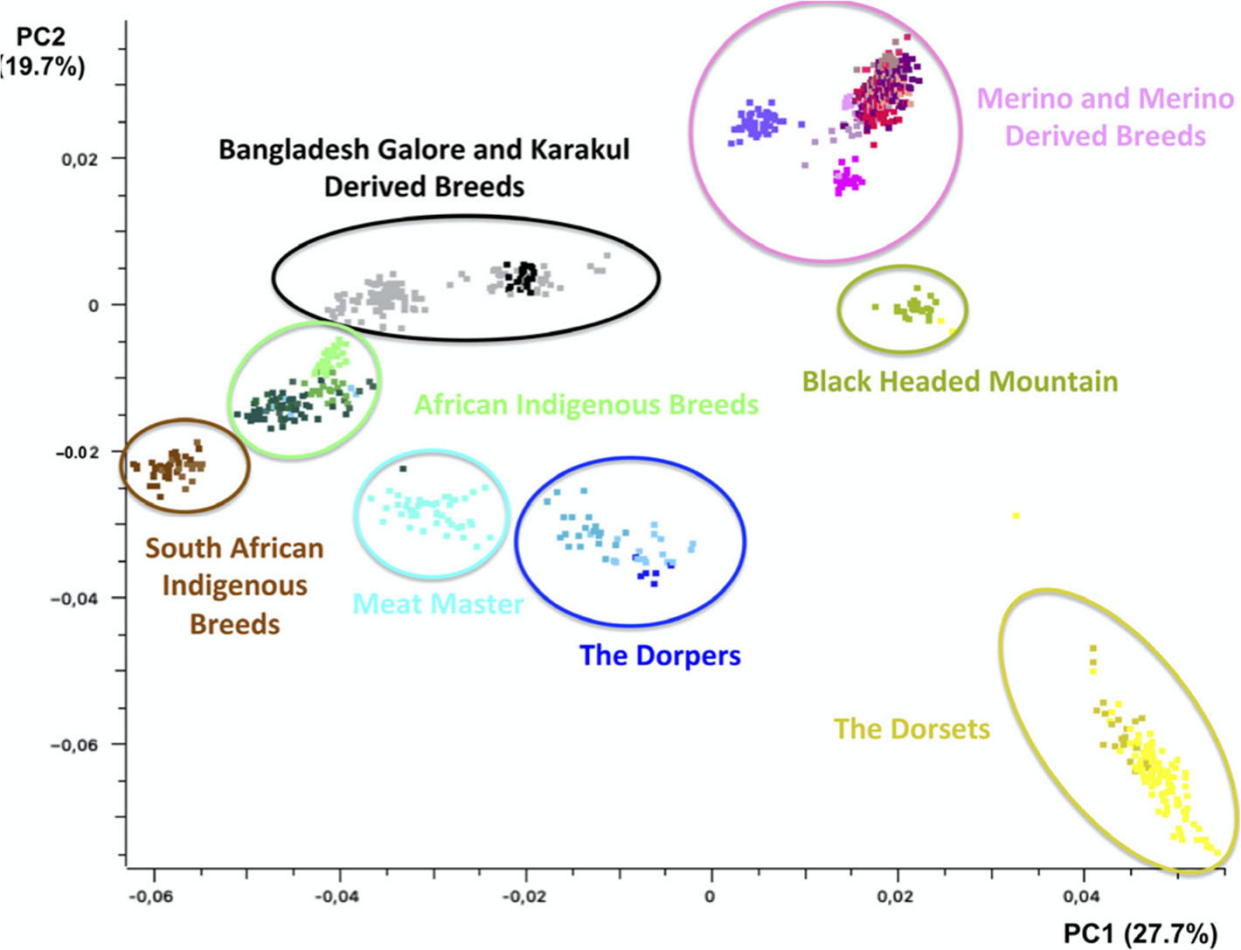
PCA based clustering of breeds (Dzomba et al., 2020)

The Ovine SNP50 BeadChip array is a genome-wide genotyping array for sheep and was developed by Illumina in collaboration with the International Sheep Genomics Consortium (ISGC). This BeadChip contains 54,241 SNPs that were chosen to be uniformly distributed across the ovine genome with an average gap size and distance of 50.9 Kb and 46 Kb, respectively, and were validated in more than 75 economically important sheep breeds (OvineSNP50 Datasheet, https://www.illumina.com/documents/products/datasheets/datasheet_ovinesnp50.pdf). This study used the Ovine SNP50 BeadChip array to investigate the distribution of ROH in South African sheep breeds sampled from different breeding goals and production systems of mutton, wool, pelt and commercial versus smallholder sectors as well as various other sheep breeds obtained globally. The objectives of the study were to investigate the occurrence and distribution of ROH; characterize autozygosity and identify genomic regions with high ROH islands with the aim to draw insights into how the South African sheep populations were in the past, as well as how their structure and demography have evolved over time. The study presumed that the founder population establishing genetic processes and the extent of breeding control have differed greatly among the different sheep breeds of South Africa and globally. This study therefore hypothesised that production systems geared towards specific traits of importance such as mutton, wool, pelt or multiple traits (as with some dual-purpose breeds) or absence of selection programs e.g. in non-descript breeds kept by smallholder farmers influences the occurrence and distribution of ROH. In a previous study [19], the South African sheep breeds clustered according to breed and production system as illustrated in Fig. 1. Using ROHs, the current study was therefore used to infer the impact of breed history, inbreeding levels and selection on the accumulation of homozygous mutations in the diverse sheep populations. Global sheep populations accessed from the ISGC (http://www.sheephapmap.org) were used to further analyse the development and separation of populations from their presumed founder populations.

## Methods

### Animal populations

Four hundred animals belonging to 14 South African breeds/populations consisting of mutton (South African Mutton Merino (*n* = 10), Dohne Merino (*n* = 50), Meatmaster (*n* = 48), Blackhead Persian (*n* = 14) and Namaqua Afrikaner (*n* = 12), pelt (Swakara subpopulations of Grey (*n* = 22); Black (*n* = 16); White-vital (*n* = 41) and White-subvital (*n* = 17) and Karakul (n = 10)); wool (SA Merino (*n* = 56), dual purpose breeds (Dorper (*n* = 23); Afrino (*n* = 51) and non-descript Nguni sheep (*n* = 30) were used in the study.

The South African Mutton Merino was developed from German Merinos and kept as a dual purpose breed for meat and wool. Dohne Merino were developed through intensive selection of merino sheep and are robust animals that are resistant and tolerant to diseases and parasites. The Dohne Merino together with the Afrino and Meatmaster are South African breeds that were developed from Merino breeds either through intensive selection as in the case of the Dohne Merino or through crossbreeding with indigenous sheep breeds of Ronderib Afrikaner for Afrino and Damara for Meatmaster [20]. The Swakara subpopulations were derived from Karakul sheep and bred and developed for pelt production, for which they are predominantly farmed in the Southern parts of Africa [21]. The Blackhead Persian are fat tailed sheep that were imported into South Africa from Somalia in 1870 and are currently farmed by smallholder farmers primarily for meat. Namaqua Afrikaner sheep are indigenous to South Africa and, like the Blackhead Persian, are also farmed by smallholder farmers. The detailed list of breeds and sample sizes are outlined in Table 1. The commercial meat and wool breeds were sampled from Grootfontein Agricultural Development Institute (GADI) biobank and other commercial farms in the Eastern Cape and Northern Cape Provinces of the country [22]. The Swakara sheep were sampled from Swakara pelt farming farms in Namibia and from the Northern Cape province of South Africa. The Nguni is a non-descript indigenous sheep of South Africa raised by communal farmers in the KwaZulu-Natal region of South Africa from where it was sampled.

**Table 1:** Mean and Standard deviation of ROH based inbreeding (F_ROH_) of South African and global sheep populations

Breed	No. animals	Mean F_**ROH**_	SD	Mean F_**HOM**_	SD
Afrino	51	0.1621	0.0212	0.1265	0.0210
African Dorper	21	0.1551	0.0376	0.2165	0.0996
African White Dorper	6	0.2011	0.0333	0.1631	0.0352
Australian Industry Merino	88	0.0914	0.0272	0.1035	0.0479
Australian Merino	50	0.1028	0.0421	0.1266	0.0381
Australian Poll Dorset	108	0.1761	0.0402	0.1171	0.0400
Australian Poll Merino	98	0.0787	0.0275	0.0491	0.0278
Blackhead Persian	14	0.3085	0.0435	0.3425	0.0434
Black Vital Swakara	20	0.2919	0.0534	0.2892	0.0515
Bangladesh Galore	24	0.2333	0.0671	0.2701	0.0628
Black-headed Mutton	24	0.1839	0.1164	0.1561	0.1202
Chinese Merino	23	0.0955	0.0489	0.0646	0.0492
Dohne Merino	50	0.1031	0.0167	0.0754	0.0180
Dorper	23	0.2374	0.0890	0.2165	0.0996
Dorset Horn	21	0.2417	0.0604	0.1927	0.0625
Ethiopian Menz	34	0.1210	0.0387	0.1789	0.0353
Grey Vital Swakara	22	0.2049	0.0588	0.1998	0.0562
Karakas	18	0.0806	0.0553	0.0947	0.0552
Meatmaster	46	0.1260	0.0202	0.1206	0.0209
MacArthur Merino	12	0.4484	0.0332	0.3505	0.1356
Merinolandschaf	22	0.1006	0.0146	0.0759	0.0153
Nguni	30	0.3138	0.0521	0.3477	0.0487
Namaqua Afrikaner (SA)	12	0.3208	0.1318	0.3129	0.0746
Namaqua Afrikaner (ISGC)	10	0.2614	0.0272	0.2218	0.0213
Red Massai	45	0.1008	0.0234	0.1694	0.0283
Ronderib Afrikaner	19	0.1971	0.0665	0.1943	0.0664
South African Merino	56	0.1404	0.0467	0.1408	0.0294
South African Mutton Merino	10	0.2237	0.0366	0.1907	0.0386
Swakara	6	0.2615	0.0364	0.2593	0.0354
White Sub-Vital Swakara	16	0.2859	0.0795	0.2803	0.0801
White Vital Swakara	40	0.2822	0.0520	0.2754	0.0505
**Overall**	**1019**	**0.1622**	**0.0900**	**0.1461**	**0.0992**

### Genotyping

#### Genotyping & SNP quality control

The 400 sheep were genotyped using the Illumina Ovine SNP50 BeadChip on the Infinium assay platform at the Agricultural Research Council-Biotechnology Platform in South Africa. SNP genotypes were called using genotyping module integrated in GenomeStudio™ V2010.1 (Illumina Inc.).

### Global sheep populations

Additional 623 genotypes from a global set of sheep breeds representing worldwide populations were included in the analysis. These populations included breeds of African (6), Asian (2) and European (9) origin. The African breeds comprised African Dorper (*n* = 21), African White Dorper (*n* = 6), Ethiopian Menz (*n* = 34), Namaqua Afrikaner (*n* = 10), Red Maasai (*n* = 45) and Ronderib Afrikaner (*n* = 19). Asian populations includedBangladesh Garole (*n* = 24) and Karakas (*n* = 18). Finally, the breeds of European origin included Australian Poll Dorset (*n* = 108), Australian Industry Merino (*n* = 88), Australian Merino (*n* = 50), Australian Poll Merino (*n* = 98), Chinese Merino (*n* = 23), MacArthur Merino (*n* = 12), Dorset Horn (*n* = 21), Merinolandschaf (*n* = 22) and Black-headed Mountain (n = 24). This data set was accessed with permission from the ISGC (http://www.sheephapmap.org).

The two data sets were merged into a dataset that consisted of 1019 animals from 31 sheep breeds/populations and 43,556 SNPs that were retained for analyses after global quality control of both the South African and ISGC sheep breeds (Table 1). Chromosomal coordinates for each SNP were obtained from ovine genome assembly 4.1 (OAR4.1). Markers were filtered to exclude loci assigned to unmapped contigs. Only SNPs located on autosomes were considered for further analyses. Moreover, the following filtering parameters were adopted to exclude certain loci and animals and to generate the pruned input file: (i) SNPs with a call rate < 95% and (ii) minor allele frequency < 1% and (iii) animals with more than 2% of missing genotypes were removed. File editing was carried out using Plink [23].

### Runs of homozygosity definition

Runs of homozygosity were computed using the R package detectRUNS and the consecutive runs method [24]. No pruning was performed based on LD, but the minimum length that constituted the ROH was set to 1 Mb to exclude short ROH deriving from LD. The following criteria were used to define the ROH: (i) one missing SNP and up to one possible heterozygous genotype was allowed in the ROH, (ii) the minimum number of SNPs that constituted the ROH was set to 30 (iii) the minimum SNP density per ROH was set to one SNP every 100 Kb and (iv) the maximum gap between consecutive homozygous SNPs was 250 Kb. The computed ROHs were then categorised into bins based on lengths of 1–6 Mb, 6–12 Mb, 12–24 Mb, 24–48 Mb and > 48 Mb.

The mean number (MN_ROH_) and average length (AL_ROH_) of ROH per breed as well as the average sum of ROH segments per breed were estimated. The inbreeding coefficient (F_ROH_) was estimated based on the ROH for each animal and averaged per breed. F_ROH_ was calculated within detectRUNS using the following formula:

$$ {F}_{ROH}={L}_{ROH}/{L}_{AUTO}, $$

where:

*L*_*ROH*_ is the total length of ROH on autosomes and;

*L*_*AUTO*_ is the total length of the autosomes covered by SNPs, which was 2453 Mb.

For comparison, inbreeding coefficients were also estimated using variance between observed and expected heterozygosity (F_HOM_). This was done using Golden Helix SVS software.

### Detection of common runs of homozygosity

To identify the genomic regions most commonly associated with ROH for the meta-population and for groups on the basis of production purposes (mutton, wool and pelt and dual purpose breeds), Golden Helix SVS was used to analyse the incidence of common runs per SNP, which was then plotted against the position of the SNP along the chromosome (OAR).

ROH islands were defined as clusters of runs that were > 1000 Kb with a minimum of 30 SNPs and found in more than 20 samples and analysed using Golden Helix SVS. For each sample, the proportion of SNPs in the ROH island was estimated. The mean proportion of SNPs per sample per ROH islands was determined using Proc MEANS procedure in SAS v9.4 [25]. The variance in mean proportion of SNPs in ROH islands amongst breeds was analysed using the Proc GLM in SAS v9.4 [25] using the following model:

*Proportion of SNPs per ROH island = μ + B*_*i*_ *+ e.*

where:

*μ = overall mean;*

*B*_*i =*_ *Breed effect and;*

*e = random residual error*

### Functional annotation of ROH islands

ROH islands that were constituted by SNPs from 1, 2 or 3 populations (considered as unique islands) and those common islands with SNPs from three quarters of the populations (> 23 populations) were used for functional annotation. The genomic region associated with each of these island was annotated using the Sheep Quantitative Trait Loci (QTL) database (https://www.animalgenome.org/cgi-bin/QTLdb/OA/summary) and the University of Carlifonia Santa Cruz (UCSC) Genome Browser (http://genome.ucsc.edu/). The genomic coordinates for these ROH islands were used for the annotation of genes that were fully or partially contained within each selected region using the UCSC Genome Browser (http://genome.ucsc.edu/) and submitted to the Database for Annotation, Visualization and Integrated Discovery (DAVID) database (http://david.abcc.ncifcrf.gov/) for gene ontology (GO). Finally, the Kyoto Encyclopedia of Genes and Genomes (KEGG) analysis was used to investigate pathways associated with each annotated gene within ROH islands. Significant enrichment in the candidate genes was indicated by a *p*-value of < 0.05.

## Results

### Runs of homozygosity counts

**Fig. 2:**
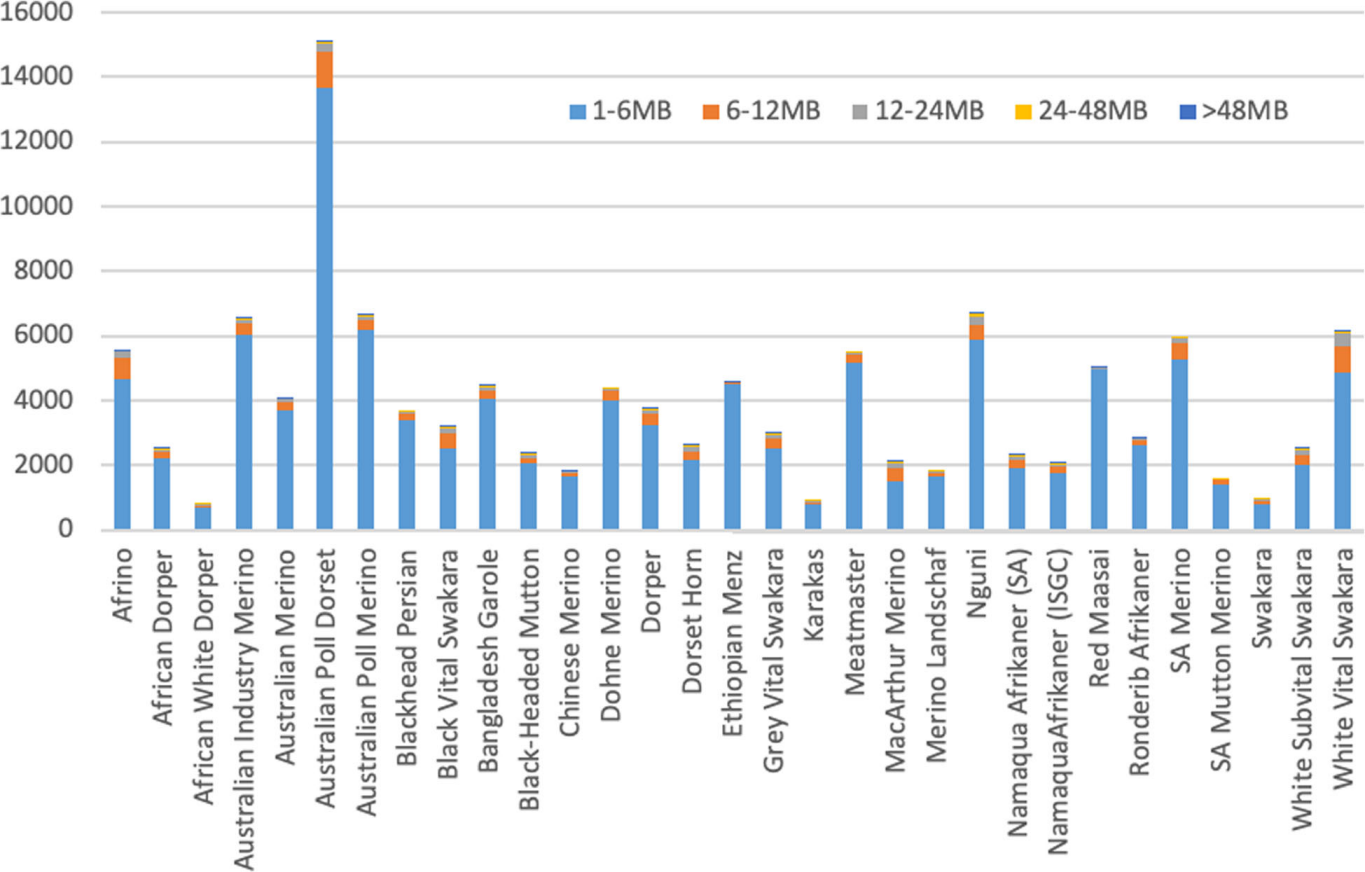
Runs of Homozygosity of different lengths per breed

The study identified 121,399 ROH in total with mean number of ROH per animal per breed ranging from 800 (African White Dorper) to 15,097 (Australian Poll Dorset) as illustrated in Fig. 2 and Supplementary Table S1. Analysis of the distribution of ROH according to their size showed that, for all breeds, the majority of the detected ROH were in the smallest 1–6 Mb in length category (88.2%) ranging from 684 in African White Dorper (*n* = 684) to Australian Poll Dorset (*n* = 13,677). The longest ROHs (> 48 MB) were the least (*n* = 108) with most animals detecting no ROH in this category. The Black Head Mountain had largest number of long (> 48 Mb) of 30 followed by Dorset Horn with 16 ROH > 48 Mb as illustrated in Fig. 2. The average length of ROH across breeds was 5.88 Mb and ranged from 2.60 Mb (Afrino) to 6.90 Mb (Nguni).

### Inbreeding coefficient

McArthur Merino showed the highest value of inbreeding on the basis of ROH (F_ROH_ = 0.45 ± 0.03), whereas Australian Poll Merino (F_ROH_ = 0.08 ± 0.03) showed the lowest (Table 1). Of the South African breeds, the Nguni and the Blackhead Persian displayed high F_ROH_ of 0.31 ± 0.05 and 0.31 ± 0.04, respectively. Other breeds with high F_ROH_ included the Black Vital Swakara and the White Subvital and Vital Swakara with F_ROH_ > 0.28 (Table 1). South African breeds with low F_ROH_ included Dohne Merino (F_ROH_ = 0.10 ± 0.02), the Meatmaster with F_ROH_ of 0.13 ± 0.02 and South African Merino with F_ROH_ = 0.14 ± 0.05. Inbreeding coefficient based on variance F_HOM_ are presented in Table 1. A correlation between F_ROH_ and F_HOM_ was observed, with breeds such as Blackhead Persian, Nguni displaying high F_ROH_ and F_HOM_, respectively.

### ROHs per chromosome per breed

**Fig. 3:**
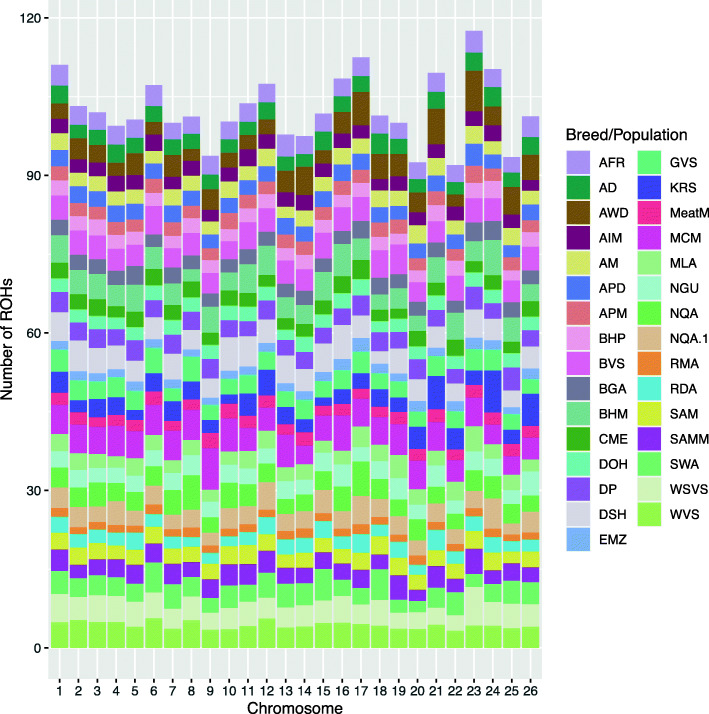
Number of ROHs per chromosome per breed. AFR = Afrino. AWD = African White Dorper. AIM = Australian Industry Merino. AM = Australian Merino . APD = Australian Poll Dorset. APM = Australian Poll Merino. BHP = Blackhead Persian. BVS = Black Vital Swakara. BGM = Bangladesh Garole. BHM Blackheaded Mountain. CME = Chinese Merino. DOH = Dohne Merino. DP = Dorper. DSH = Dorset Horn. EMZ = Ethiopian Menz. GVS = Grey Vital Swakara. KRS = Karakas. MeatM = Meatmaster. MCM = MacArthur Merino. MLA = Merinolandscha. NGU = Nguni. NQA = Namaqua Afrikaner. RMA = Red Maasai. RDA = Ronderib Afrikaner. SAM = SA Merino. SAMM = SA Mutton Merino. SWA = Swakara=. WSVS = White Subvital Swakara. WVS = White Vital Swakara

The distribution of ROHs per chromosome per breed are illustrated in Fig. 3. Runs were evenly distributed amongst chromosomes within breeds.

### Incidences of common runs per SNP

**Fig. 4:**
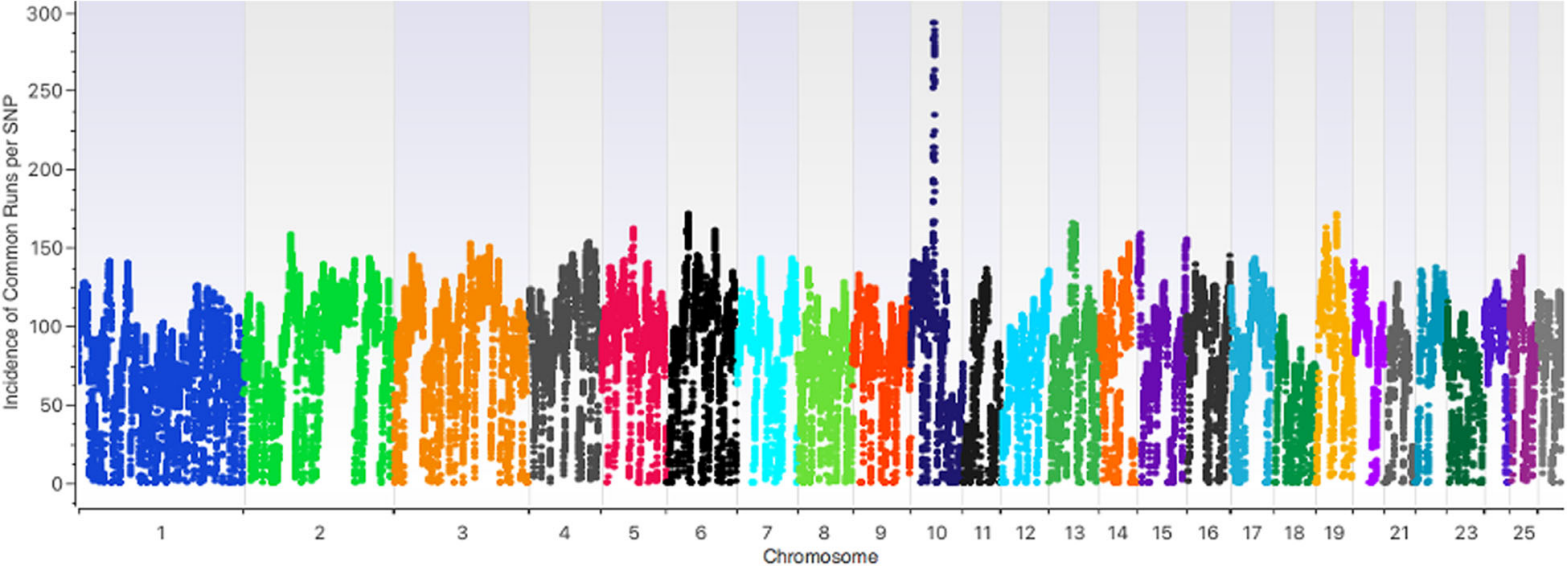
Incidences of common runs per SNP per chromosome

Using Golden Helix SVS, an analysis was conducted to investigate the incidence of common runs per SNP and results are illustrated in Fig. 4 and Supplementary Table S2. Highest incidence of common runs per SNP across breeds was observed on chromosome 10 with over 250 incidences of common ROHs at some of the SNPs (Fig. 4; Supplementary Table S2). Other chromosomes such as 2, 6, 13, 15 and 19 were found to have moderate incidences of common SNPs averaging 150–160 (Fig. 4). Across breeds, certain regions were observed to be absent of ROHs notable of which were chromosomes 10 (±7Mbs region; 21 (±40Mbs region); 22 (±18Mbs region) and 26 (±8Mbs region) as illustrated in Supplementary File S3).

### ROH islands

A total of 244 ROH islands distributed across all 26 autosomes were observed. Mean proportion of SNPs in ROH island ranged from 0.02 ± 0.15 (island ROH224 on chromosome 23) to 0.13 ± 0.29 (island ROH175 on chromosome 15) as illustrated in Supplementary Table S4. Number of islands ranged from a minimum of 2 clusters per chromosome (on chromosome 22) to 32 clusters per chromosome (on chromosome 1). Seventeen (17) of the islands were observed in single populations and considered unique ROH islands. Thirty-nine of the reported ROHs were each observed in 3 populations whilst the remaining 188 were each observed in more than 3 populations and considered common islands. Detailed distribution of ROH islands are presented in Supplementary Table S5 and Supplementary File S6a-d. The MacArthur Merino population had five unique ROH islands (Supplementary File S6b) followed by Nguni (Supplementary File S6b) and Blackhead Persian (Supplementary File S6c) with three each whilst the South African Merino, South African Mutton Merino, White Vital Swakara, Karakul, Dorset Horn and Chinese Merino each had one unique ROH island (Supplementary File S6d). For those islands shared between two populations, the Blackhead Persian shared with South African Mutton Merino, SA Merino, Black Vital Swakara, Ronderib Afrikaner and MacArthur Merino; the MacArthur Merino shared with Nguni, Namaqua Afrikaner, Blackhead Persian and Bangladesh Galore; the Nguni shared with MacArthur Merino, White Vital Swakara and Karakul, the Dorper with NQA and RDA while the AWD shared with South African Mutton Merino. For ROH islands shared amongst 3 populations the Nguni shared one with White Vital Swakara and Karakul; the African White Dorper one with Namaqua Afrikaner and White Subvital Swakara; the Bangaldesh Galore with Blackhead Persian and South African Mutton Merino; the Dorset Horn with Nguni and White Vital Swakara while the BVS with RDA and South African Mutton Merino.

### SNPs and gene annotation

Based on the Sheep QTL database, ROH7 on chromosome one, and unique to Blackhead Persian lies within a genomic region previously found to be harboring QTLs for selection for presence or absence of horns in Soay sheep and average daily gain in Awassi and Merino sheep. ROH island ROH15 on chromosome one and unique to MacArthur Merino lies in a genomic region harboring QTLs for Feacal Egg Count and Susceptibility to facial eczema. QTLs associated with carcass fat percentage (*FATP*) in Awassi and Merino sheep were also observed within island (ROH15).

A list of genes and associated KEGG pathways found with population restricted ROH islands are reported in Table 2a and b and Supplementary Table S5. Using the KEGG pathway analysis, genes associated with metabolic pathways such as *ANPEP, HDDC3 and ST3GAL3* where observed with ROH islands unique to the Blackhead Persian (Table 2a). Two ROH islands (ROH100 and ROH101) unique to Blackhead Persian and Nguni respectively were observed in chemokine pathways (*GRK4 gene*) and Natural killer cell mediated cytotoxicity pathways (*SH3BP2 gene*). Other pathways observed included thermogenesis (ROH199), interleukin signalling pathways (ROH11) and pathways associated with bacterial infections such as *Escherichia Coli, Salmonella, Mycobacterium tuberculosis* (ROH149) as illustrated in Table 2.

**Table 2:** ROH island observed in < 3 populations and the associated genes and KEGG pathways

**ROH ISLAND**	**CHR**	**BREEDS**	**GENES**	**KEGG PATHWAY**
ROH2	1	BHP	*ST3GAL3.*	Metabolism (sphingolipid metabolism).
ROH199	18	BHP	*BTBD1*	Fanconi anaemia pathway
			*WHAMM*	Tight junction
			*BLM*	Fanconi anaemia pathway
			*FES*	Biosynthesis of secondary metabolites
			*FANCI*	Fanconi anaemia pathway
			*PLIN1*	Regulation of lipolysis in adipocytes; Thermogenesis
			*PEX11A*	Peroxisome
			*ANPEP*	Renin-angiotensin system; Hematopoietic cell lineage; Metabolic pathways
			*HDDC3*	Purine metabolism; Metabolic pathways
ROH100	6	BHP	*ADRA2C*	CGMP-PKG signalling pathway
			*GRK4*	Chemokine signalling pathway; Endocytosis
			*SH3BP2*	Natural killer cell mediated cytotoxicity
			*PIGG*	Prodigiosin biosynthesis; Metabolic pathways
ROH101	6	NGU	*ADRA2C*	CGMP-PKG signalling pathway
			*GRK4*	Chemokine signalling pathway; Endocytosis
			*SH3BP2*	Natural killer cell mediated cytotoxicity
			*PIGG*	Prodigios in biosynthesis; Metabolic Pathways
ROH108	7	SAM	*DAAM1*	Wnt signalling pathway
			*L3HYPDH*	Arginine and proline metabolism
ROH149	12	MCM	*IL10*	Cytokine-cytokine interaction; Toxoplasmosis; Tuberculosis; Various diseases
ROH11	1	NGU & MCM	*PRR4*	Circadian rhythm – plant
			*S100A9*	IL-17 signalling pathway
			*CRTC2*	Glucagon signalling pathway, Insulin resistance
			*RAB13*	Tight junction
			*TPM3*	Cardiac muscle contraction
			*IL6R*	Viral protein interaction with cytokine and cytokine receptor
ROH51	3	NQA & MCM	*TPRN*	Type I diabetes mellitus
			*VAV2*	Rap1 signalling pathway
			*SARDH*	Glycine, serine and threonine metabolism
			*ABO*	Metabolic Pathways
ROH58	3	BHP & RDA	*FSHR*	Ovarian steroidogenesis
			*LHCGR*	Prolactin signalling pathway
ROH75	4	BHP & MCM	*VPS41*	Salmonella infection; Autophagy - yeast
			*AMPH*	Inflammatory mediator regulation of TRP channels; Biosynthesis of secondary metabolites; Metabolic Pathways; Fc gamma R-mediated phagocytosis
			*WNT16*	Signalling pathways regulating pluripotency of stem cells; Melanogenesis
ROH125	9	BHP & SAM	*AASS*	Lysine degradation; Metabolic pathways Biosynthesis of secondary metabolites;
			*MSC*	Sulfur metabolism; Degradation of aromatic compounds; Microbial metabolism in diverse environments; Metabolic pathways; *Staphylococcus aureus* infection
			*TRPA1*	Inflammatory mediator regulation of TRP channels
			*RDH10*	Retinol metabolism
			*UBE2W*	Ubiquitin mediated proteolysis
			*LY96*	NF-kappa B signalling pathway; Toll-like receptor signalling pathway; Salmonella infection; Pertussis; Toxoplasmosis
ROH200	18	BHP & SAMM	*CTSH*	Lysosome; Apoptosis
			*RASGRF1*	Ras signalling pathway; MAPK signalling pathway; Focal adhesion
			*BCL2A1*	NF-kappa B signalling pathway; Apoptosis
			*FAH*	Microbial metabolism in diverse environments; Metabolic pathways; Nitrogen metabolism
			*IL16*	Cytokine-cytokine receptor interaction
			*MCEE*	Carbon metabolism; Metabolic pathways; Microbial metabolism in diverse environments; Valine, leucine and isoleucine degradation
			*FAN1*	Fanconi anaemia pathway
ROH11	1	NGU & MCM	*S100A9*	IL-17 signalling pathway
			*S100A8*	IL-17 signalling pathway
			*BGLAP*	Parathyroid hormone synthesis, secretion and action
			*ARHGEF2*	Pathogenic *Escherichia coli* infection; Tight junction; Bacterial invasion of epithelial cells; Salmonella infection
			*ASH1L*	Metabolic pathways; Lysine degradation
			*FDPS*	Biosynthesis of secondary metabolites; Metabolic pathways
ROH232	24	BVS; RDA; SAMM	*STUB1*	Protein processing in endoplasmic reticulum; Ubiquitin mediated proteolysis
			*SSTR5*	Growth hormone synthesis, secretion and action; cAMP signalling pathway
			*UBE2I*	Ubiquitin mediated proteolysis; RNA transport
			*GNPTG*	Lysosome
			*ORAI2*	Calcium signalling pathway
			*POR*	RNA transport; ABC transporters; Protein digestion and absorption; Carbohydrate digestion and absorption; Glycolysis / Gluconeogenesis

*BHP* Blackhead Persian, *Ngu* Nguni, *MCM* MacArthur Merino, *SAM* SA; Merino, *NQA* Namaqua Afrikaner, *RDA* Ronderib Afrikaner, *NQA* Namaqua Afrikaner, *RDA* Ronderib Afrikaner, *SAMM* SA Mutton Merino

## Discussion

The domestication of sheep was a complex process that allowed both natural and artificial selection of breeds. Regional variations due to genetic drift in breeds became small and geographically restricted resulting in extensive and diverse phenotypes. Such processes, while well documented in other breeds, are unknown in most of the small and geographically restricted local populations. ROH have been extensively studied in humans and livestock populations and are an established method of inferring population history. ROH are continuous homozygous segments that are common in individuals and populations. The ability of these homozygous segments to give insight into a population’s genetic events makes them a useful tool that can provide information about the demographic evolution of a population over time. Furthermore, ROH provide useful information about the genetic relatedness among individuals, helping to manage inbreeding rate, thereby exposing possible deleterious variants in the genome. ROH are widely used as predictors of inbreeding levels in populations. Calculating the inbreeding coefficient from ROH (F_ROH_) is more accurate for estimating autozygosity and for detecting both past and more recent inbreeding effects than estimating inbreeding from pedigree data [26].

South African sheep populations are a result of complex and multifaceted production systems. Natural and artificial selection forces play vital roles through mixtures of indigenous, commercial and synthetic/composite breeds raised in extreme production conditions. ROH were used in this study to investigate this population history with emphasis on breed relatedness. Illumina ovine SNP50 genotypes of South African mutton, wool, pelt and dual purpose and non-descript breeds was analysed together with that of global populations of similar geographic background.

Breeds such as the Australian Poll Dorset followed by the South African Nguni, Australian Poll Merino and Australian Industry Merino and the White Vital Swakara sheep had the most ROHs ranging from 6155 in White Vital Swakara to 15,097 in Australian Poll Dorset. Frequency of ROHs in different breeds reflect on the size of the breeds often positively associated with inbreeding levels [27]. South African breeds of the Nguni, Blackhead Persian, Namaqua Afrikaner and Swakara are small breeds restricted to specific production systems and geographic locations [28, 29], which explains the high frequency of ROH in these populations. Similarly-raised worldwide populations include the Ethiopian Menzi [30, 31], Bangladesh Galore [32], Black-headed Mutton [33] that also had high numbers of ROH in this study. ROH due to recent inbreeding tends to be longer, due to little opportunity for recombination to break up the segments that are identical-by-descent [34]. The Nguni (725) Afrino (845), White Vital Swakara (1204) and Australian Poll Dorset (1359) had the most moderately sized (6–24 Mb) ROH while the Australian Poll Dorset (61), DSH (75), BHM (90), Nguni (90) and White Vital Swakara (94) has the most of the large (> 24 Mb) ROH (Supplementary Table 1) presenting more ancient inbreeding. Ancient ROH are generally much shorter because the chromosomal segments have been broken down by repeated meiosis [27, 34]. In this study, the Nguni (5855), Australian Industry Merino (6040), Australian Poll Merino (6188) and Australian Poll Dorset (13,677) had the highest number of short ROH (Supplementary Table 1) implying more recent inbreeding events. The Nguni breed of South Africa is a non-descript breed kept by smallholder farmers under low-input communal farming systems [29, 35]. Small flock sizes, sharing and retaining of bucks for multiple breeding cycles characterises the smallholder livestock production systems of South Africa inclusive of sheep. It has been suggested that such production factors lead to inbreeding in these populations [36].

Notable inbreeding has been observed in Zulu sheep [29, 36]. The spread of Zulu sheep into different areas of KwaZulu-Natal has fractured the sheep into isolated subpopulations occupying different ecological, social-cultural and management environments [20]. The high frequency of both short and long ROH in the Nguni relative to other breeds is supported by the breed history and suggestive of a sustained high level of inbreeding due to both founder effects and the practice by smallholder farmers of raising the sheep as small fragmented populations. Contrary to the Nguni are the composite commercial breeds of South Africa, e.g. Afrino, Meatmaster, that, although they experienced population bottlenecks during their formation which is now reflected by the high frequency of short ROH, the breeds are now well managed commercially and as such present minimum long ROH > 24 Mb. The Blackhead Persian were initially introduced to South Africa by chance in 1869. A vessel damaged by a storm at sea carried a number of slaughter sheep. These sheep, one ram and three ewes, were taken to Wellington where the breed was further developed [21]. In 1948, the present Blackhead Persian Sheep Breeders’ Society of South Africa was formed in De Aar and to date the Black-headed Persian is represented by a well-established breed society with breed standards and management practise. Such history of the Blackhead Persian explain the predominating short ROHs reflective of ancient founder effects during its establishment in South Africa and the few long ROHs since it is now well managed with a representing breed society.

Overall, the patterns of distribution of ROH revealed in this study showed peculiar patterns of inbreeding of sheep breeds that corresponded with levels of selection pressure typical of trait of economic importance as well as the production system typical of their rearing. South African indigenous and local breeds of Nguni, Blackhead Persian, Namaqua Afrikaner and the pelt based subpopulations of the Swakara Sheep had high inbreeding estimates reflective of the small and fragmented populations. The Nguni and Namaqua Afrikaner for example are raised as small household flocks in geographically marginalised regions of the country [29] whereas the pelt based Swakara subpopulations are small populations raised for a unique production system of pelt [37]. Challenges of small effective population size and inbreeding have been suggested in these populations [29, 36, 38]. Such high inbreeding levels imply that the breeds are of low genetic diversity and at risk of extinction. Conservation efforts are therefore required to minimise further loss of genetic diversity and extinction of some of these breeds. Phenotypic and genetic characterisation as well as sustainable utilisation of the genetic resources and implementation of structured and tailor-made breeding programs have been suggested as alternative approaches to conservation of threatened genetic diversity [2]. The global populations, the MCM from south west Europe [39] had a high F_ROH_ similar to that of the Swakara subpopulations and the Namaqua Afrikaner. The Dorper, Dorset Horn and South African Mutton Merino also shared a high F_ROH_ as the Swakara breeds, MCM and Namaqua Afrikaner.

ROH frequencies vary widely within and across chromosomes [34]. Chromosome 10 had the highest incidence of common runs per SNP across breeds with over 250 incidences of common ROHs at some of the SNPs. Chromosome 10 harbours a genomic region associated with horns in sheep [39]. One of the regions on chromosome 10 harbours the *RXFP2* gene which has been reported as a main candidate for horns in Soay sheep [40]. Other studies have identified *RXFP2* within a quantitative trait locus for horn size and highly heritable in sheep [41]. Recently, it was reported a 1.8 Kb insertion in the 3′-UTR of *RXFP2* to be associated with polledness in sheep [42]. Presence and absence of horns and subsequently horn size have been the main parameters under selection in sheep pre-and post-domestication. The *RXFP2* region has been observed to be under selection in some African sheep populations [43]. Other regions on chromosome 10 were SNPs within genes such as *Crystallin lambda 1(CRYL1)* which is associated with metabolic pathways particularly pentose and glucuronate interconversions and has been observed to be under trans-specific signatures of domestication in sheep and goats [44].

Chromosome 6 also harboured SNPs with high incidence of common ROH across breeds. Some of the associated genes included the *Secreted phosphoprotein 1* (*SPP1*) and *ligand dependent nuclear receptor corepressor-like* (*LCORL*) which are on a domain of 36.15–38.56 Mb and play an essential role in tissue and embryonic growth [45, 46]. *LCORL* was observed to be associated with height in cattle [47, 48] and observed to be under selection in different sheep breeds [45, 49] and in other species [50–54]. La et al., [46] observed significantly high expression of *SPP1* in the kidney of Hu sheep. The *Prostaglandin f2-alpha synthase (PGFS)* gene on chromosome 13 is associated with the Arachidonic acid metabolism and other metabolic pathways and were observed to be associated with wool growth regulation in Aohan fine wool sheep [55].

The failure to observe ROHs in certain genomic regions (i.e Chromosomes 10, 21, 22 and 26) could be attributed to gaps in marker coverage. According to Nandolo et al. [56], artefacts due to structural variants and gaps in marker coverage could influence the screening of ROHs. However, whilst this will be considered a SNP chip effect, affecting the screening of ROH similarly across breeds, the impact of such an artefacts might have minimal effects on the breed comparisons undertaken in this study.

In this study, ROH islands were defined as clusters of runs that were > 1000 Kb with a minimum of 30 SNPs and found in more than 20 samples which represented 1.95% of the total population in this study. In other studies, a threshold of 1% was used [57]. According to Zhang et al., [58], ROH patterns are not randomly distributed across the genomes, and are seen to be distributed and shared among individuals as a result of selection events. The 244 ROH islands observed in this study varied from those with SNPs unique to one population (17 ROH islands), two-three populations (39 islands) and those distributed in more than 3 populations (188 islands). As expected, the highly inbred populations of the MCM, Blackhead Persian and Nguni had the highest frequency of unique ROHs suggestive of small, fragmented populations with small effective population sizes and evolving independently from other populations. The Nguni and Blackhead Persian for example are small breeds kept by smallholder farmers in unique productionn systems [29]. The MCM population is also a highly inbred population from west Europe [58]. The other breeds such as White Vital Swakara and Chinese Merino are also equally small breeds highly selected for specific production purposes, e.g. pelt production in the case of White Vital Swakara sheep [59]. Previous studies suggested that ROH islands are a result of intensive selection often found in populations of finite size [57, 60].

Based on the Sheep QTL database, both productive traits, i.e. average daily gain and carcass fat percentage, and adaptive traits, e.g. absence of horns, feacal egg count and susceptibility to facial eczema, were observed within reported ROH islands. The Blackhead Persian is a polled breed with both sexes lacking horns. The observed unique island, ROH7, associated with selection of absence of horns [40] will therefore be in line with ancient selection for horns in this breed. The identified regions under ROH islands associated with metabolic pathways (e.g *ST3GAL3* on island ROH2*; FES* gene on island ROH199*)*, adaptive and innate immunity (*ADRA2C, GRK4* and *SH3BH2* genes on island ROH100), thermogenesis (*PLIN1* gene on ROH199*)* relate to key traits relevant to the Blackhead Persian’s and other livestock’s survival in harsh compromised environments smallholder populations of South Africa [61]. Similarly the Nguni sheep shared genomic regions (i.e *ADRA2C, GRK4* and *SH3BH2* genes on island ROH101) as well as traits affected by different genomic regions such as island ROH11 on chromosome 1 that harboured the *S100A9* gene associated with the *IL-17* signalling pathway and *CRTC2* gene associated with the Glucagon signalling pathway. ROH islands that were shared between populations implied common selection pressures between/amongst affected breeds for example the Blackhead Persian and the South African Mutton Merino and South African Merino that shared ROH islands associated with metabolic and immune response pathways.

## Conclusions

The study reported frequency and distribution of ROHs in South African sheep breeds, relative to global populations. The pattern of distribution of ROH corresponded to breed history and production system under which they are raised. Similarities in frequency and patterns of ROHs between South African breeds and other global breeds was observed especially when comparing the Merino-type breeds. The study showed peculiar patterns of inbreeding of sheep breeds that corresponded with levels of selection pressure typical of traits of economic importance as well as the production system aligned to their rearing.

## Supplementary Information

**Additional file 1.** (CSV 1 kb)

**Additional file 2.** (CSV 10 kb)

**Additional file 3.**

**Additional file 4.**

**Additional file 5.**

**Additional file 6.**

## Data Availability

The SNP genotypes of the South African sheep breeds generated for this project are available on https://osf.io/ceup6/?view_only=a1959659de5f4d5d9bbb1c607b2d83b6 and can be downloaded upon request.

## Abbreviations

AFR: Afrino
AIM: Australian Industry Merino
AL_ROH_: Average length of ROH
AM: Australian Merino
APD: Australian Poll Dorset
APM: Australian Poll Merino
AWD: African White Dorper
BHP: Blackhead Persian
BGM: Bangladesh Garole
BHM: Blackheaded Mountain
BVS: Black Vital Swakara
CME: Chinese Merino
DOH: Dohne Merino
DP: Dorper
DSH: Dorset Horn
EMZ: Ethiopian Menz
F_HOM_: Inbreeding coefficient based on variance in expected heterozygosity
F_ROH_: Runs of homozygosity based Inbreeding coefficient
GADI: Grootfontein Agricultural Development Institute
GO: Gene ontology
GVS: Grey Vital Swakara
ISGC: International Sheep Genomics Consortium
KEGG: Kyoto encyclopaedia of genes and genomes
KRS: Karakas
LD: Linkage disequilibrium
*L*_*ROH*_: Total length of ROH on autosomes
*L*_*AUTO*_: Total length of the autosomes
Mb: Megabases
MCM: MacArthur Merino
MeatM: Meatmaster
MLA: Merinolandschaf
MN_ROH_: Mean number of ROH
NGU: Nguni
NQA: Namaqua Afrikaner
Proc GLM: Procedure Generalised Linear Model
QTL: Quantitative Trait Loci
RDA: Ronderib Afrikaner
RMA: Red Maasai
ROH: Runs of Homozygosity
SAM: SA Merino
SAMM: SA Mutton Merino
SNPs: Single Nucleotide Polymorphisms
SAS: Statistical Analysis System
SWA: Swakara
WSVS: White Subvital Swakara
WVS: White Vital Swakara
