# Supplementary material for: Runs of homozygosity analysis of South African sheep breeds from various production systems investigated using OvineSNP50k data
Source: BMC Genomics. 2021 Jan 6;22:7. doi: 10.1186/s12864-020-07314-2 (PMC7788743; doi:10.1186/s12864-020-07314-2)
Supplement: Supplementary file 3 — Additional file 3. [file 12864_2020_7314_MOESM3_ESM.pdf]

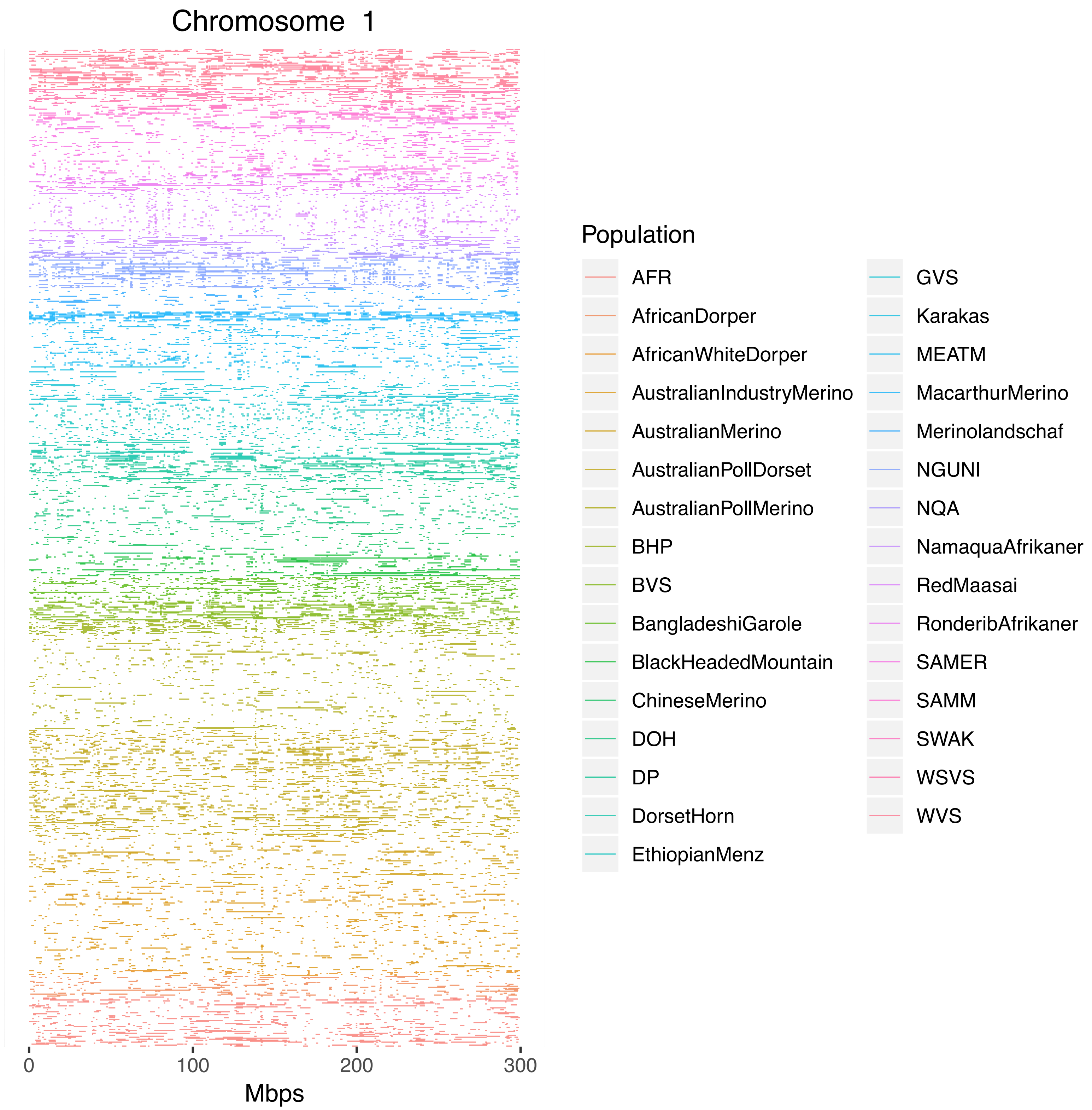

Chromosome 1
0
100
200
300
Mbps
Population
AFR
AfricanDorper
AfricanWhiteDorper
AustralianIndustryMerino
AustralianMerino
AustralianPollDorset
AustralianPollMerino
BHP
BVS
BangladeshiGarole
BlackHeadedMountain
ChineseMerino
DOH
DP
DorsetHorn
EthiopianMenz
GVS
Karakas
MEATM
MacarthurMerino
Merinolandschaf
NGUNI
NQA
NamaquaAfrikaner
RedMaasai
RonderibAfrikaner
SAMER
SAMM
SWAK
WSVS
WVS

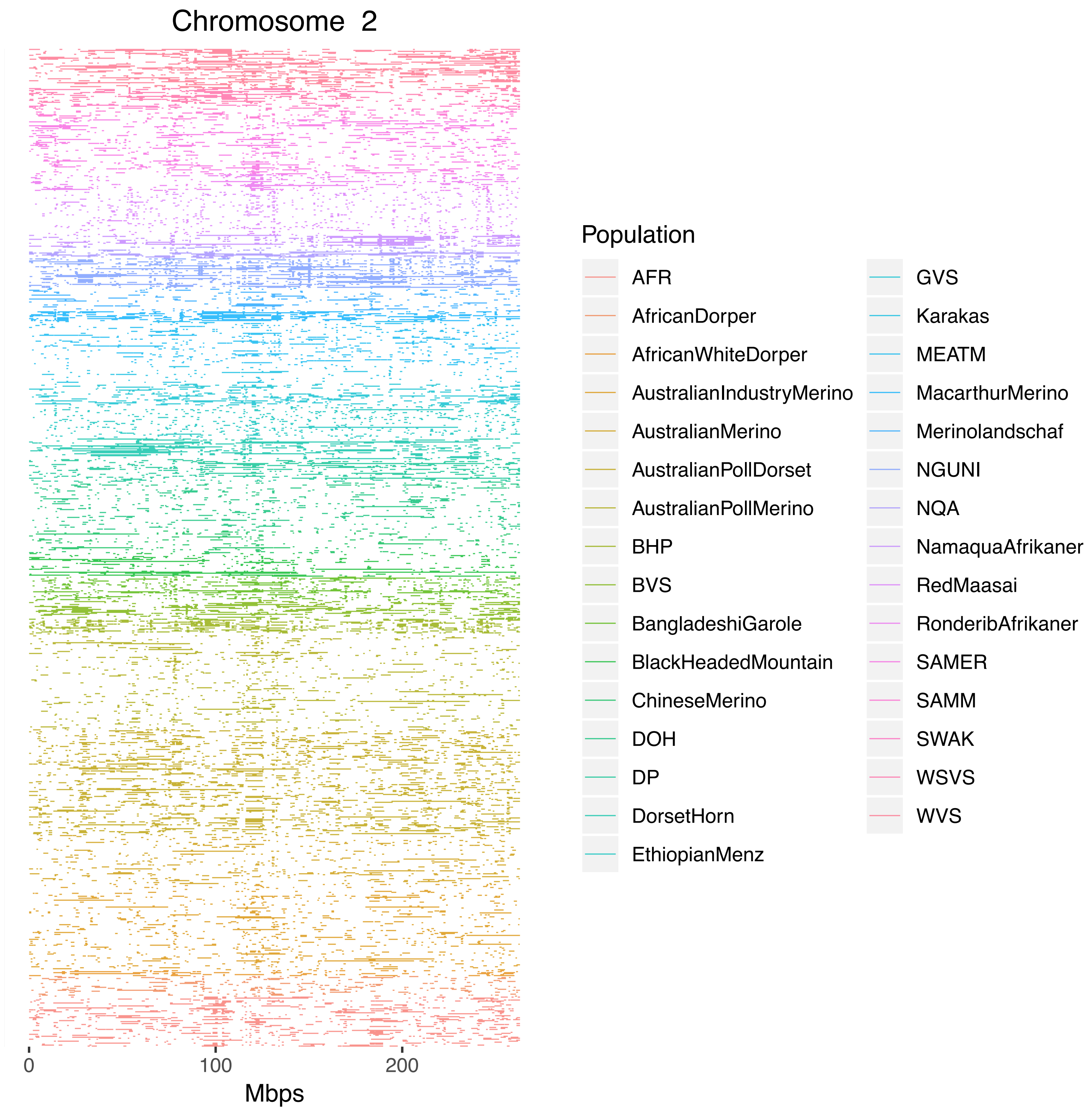

Chromosome 2
0
100
200
Mbps
Population
AFR
AfricanDorper
AfricanWhiteDorper
AustralianIndustryMerino
AustralianMerino
AustralianPollDorset
AustralianPollMerino
BHP
BVS
BangladeshiGarole
BlackHeadedMountain
ChineseMerino
DOH
DP
DorsetHorn
EthiopianMenz
GVS
Karakas
MEATM
MacarthurMerino
Merinolandschaf
NGUNI
NQA
NamaquaAfrikaner
RedMaasai
RonderibAfrikaner
SAMER
SAMM
SWAK
WSVS
WVS

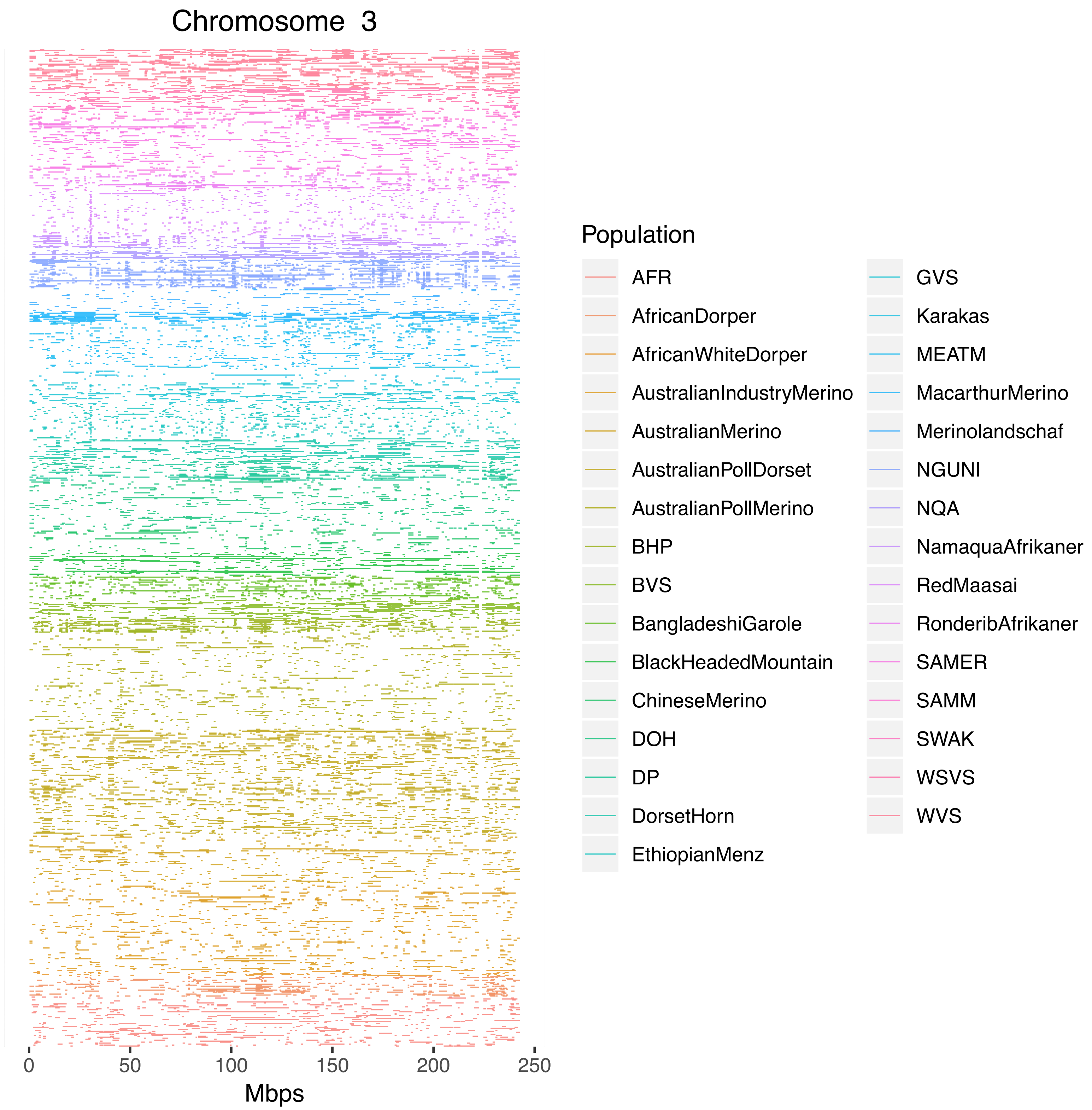
Chromosome 3
0
50
100
150
200
250
Mbps
Population
AFR
AfricanDorper
AfricanWhiteDorper
AustralianIndustryMerino
AustralianMerino
AustralianPollDorset
AustralianPollMerino
BHP
BVS
BangladeshiGarole
BlackHeadedMountain
ChineseMerino
DOH
DP
DorsetHorn
EthiopianMenz
GVS
Karakas
MEATM
MacarthurMerino
Merinolandschaf
NGUNI
NQA
NamaquaAfrikaner
RedMaasai
RonderibAfrikaner
SAMER
SAMM
SWAK
WSVS
WVS

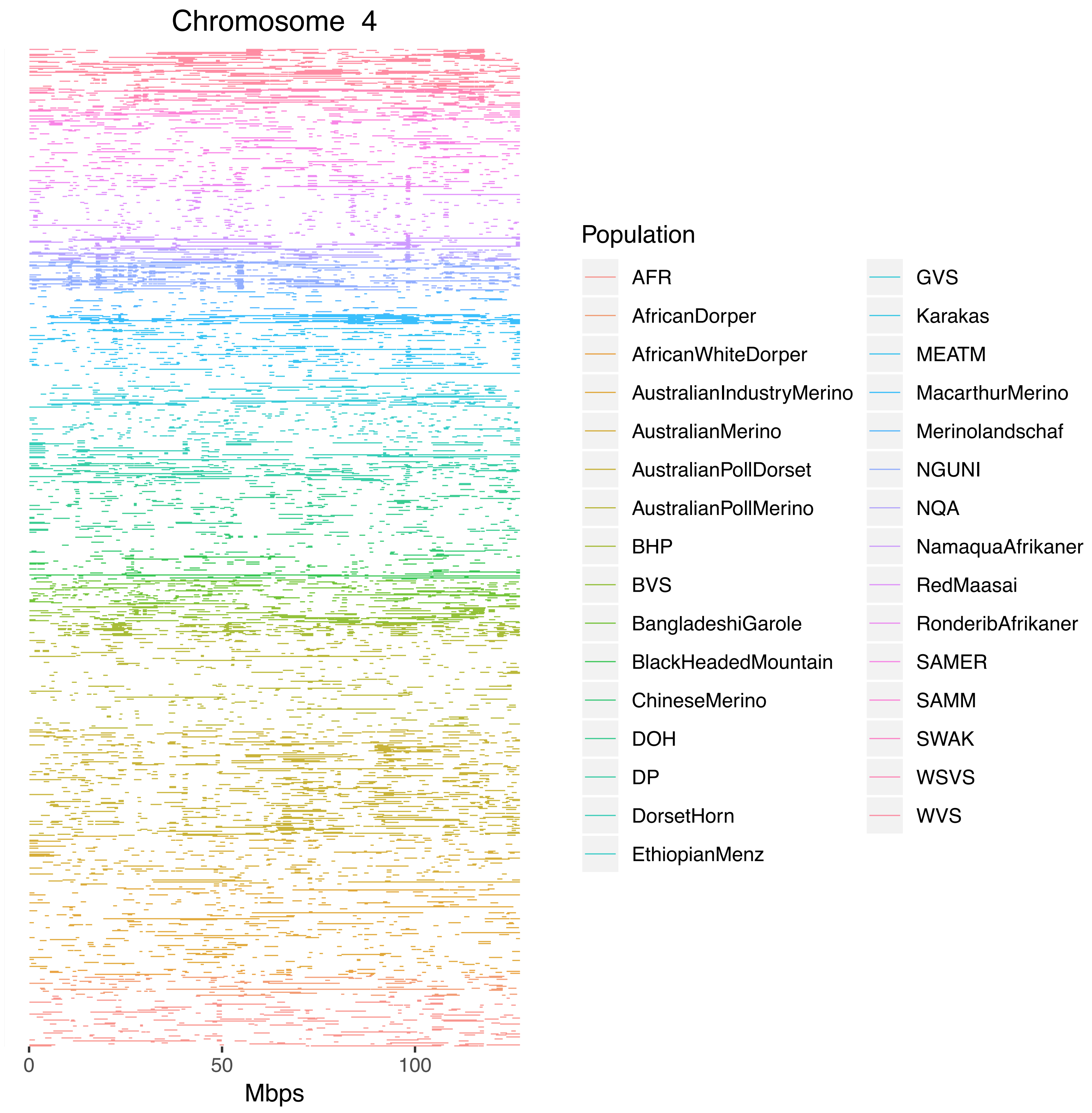

Chromosome 4
0
50
100
Mbps
Population
AFR
AfricanDorper
AfricanWhiteDorper
AustralianIndustryMerino
AustralianMerino
AustralianPollDorset
AustralianPollMerino
BHP
BVS
BangladeshiGarole
BlackHeadedMountain
ChineseMerino
DOH
DP
DorsetHorn
EthiopianMenz
GVS
Karakas
MEATM
MacarthurMerino
Merinolandschaf
NGUNI
NQA
NamaquaAfrikaner
RedMaasai
RonderibAfrikaner
SAMER
SAMM
SWAK
WSVS
WVS

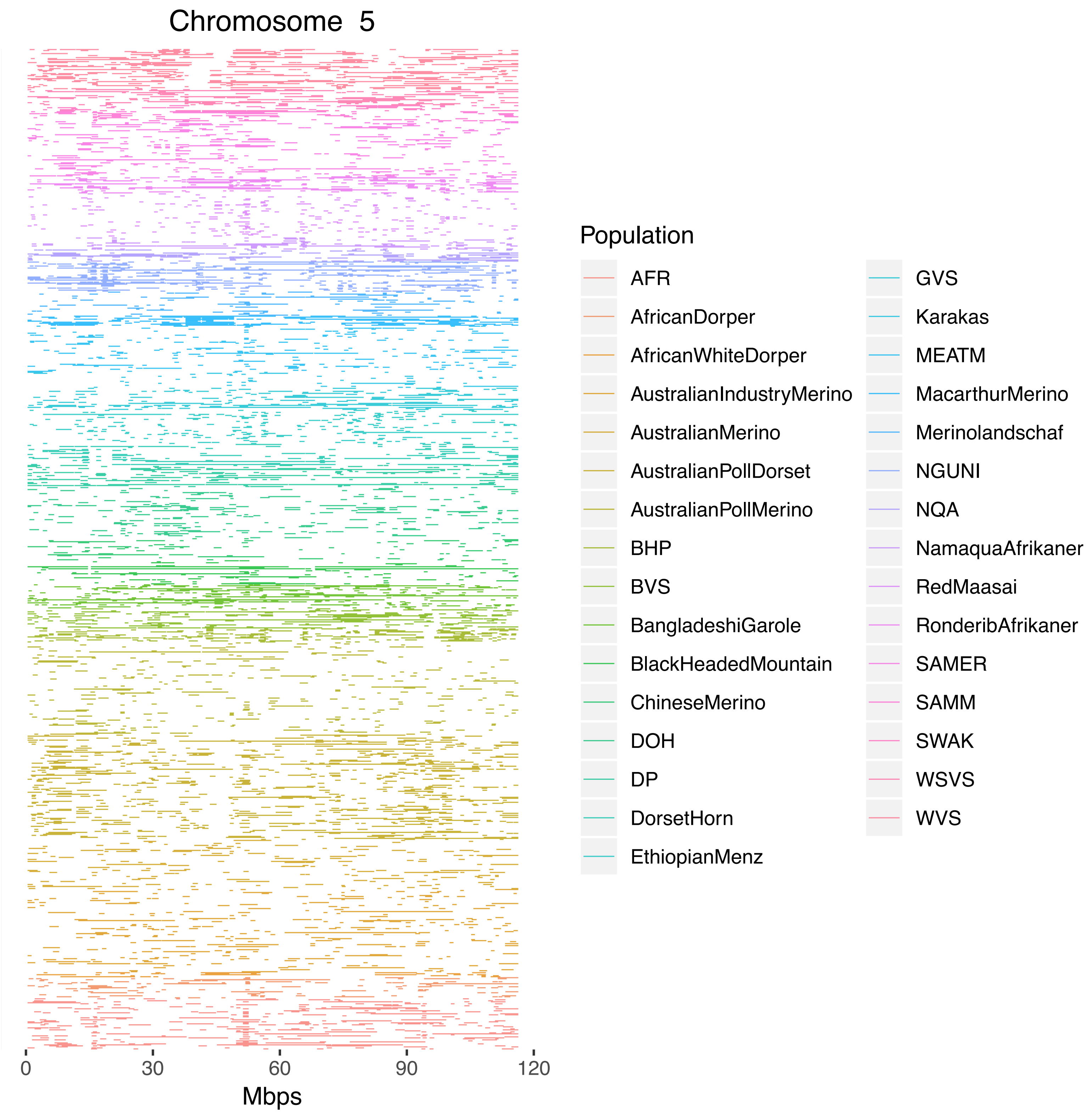

Chromosome 5
0
30
60
90
120
Mbps
Population
AFR
AfricanDorper
AfricanWhiteDorper
AustralianIndustryMerino
AustralianMerino
AustralianPollDorset
AustralianPollMerino
BHP
BVS
BangladeshiGarole
BlackHeadedMountain
ChineseMerino
DOH
DP
DorsetHorn
EthiopianMenz
GVS
Karakas
MEATM
MacarthurMerino
Merinolandschaf
NGUNI
NQA
NamaquaAfrikaner
RedMaasai
RonderibAfrikaner
SAMER
SAMM
SWAK
WSVS
WVS

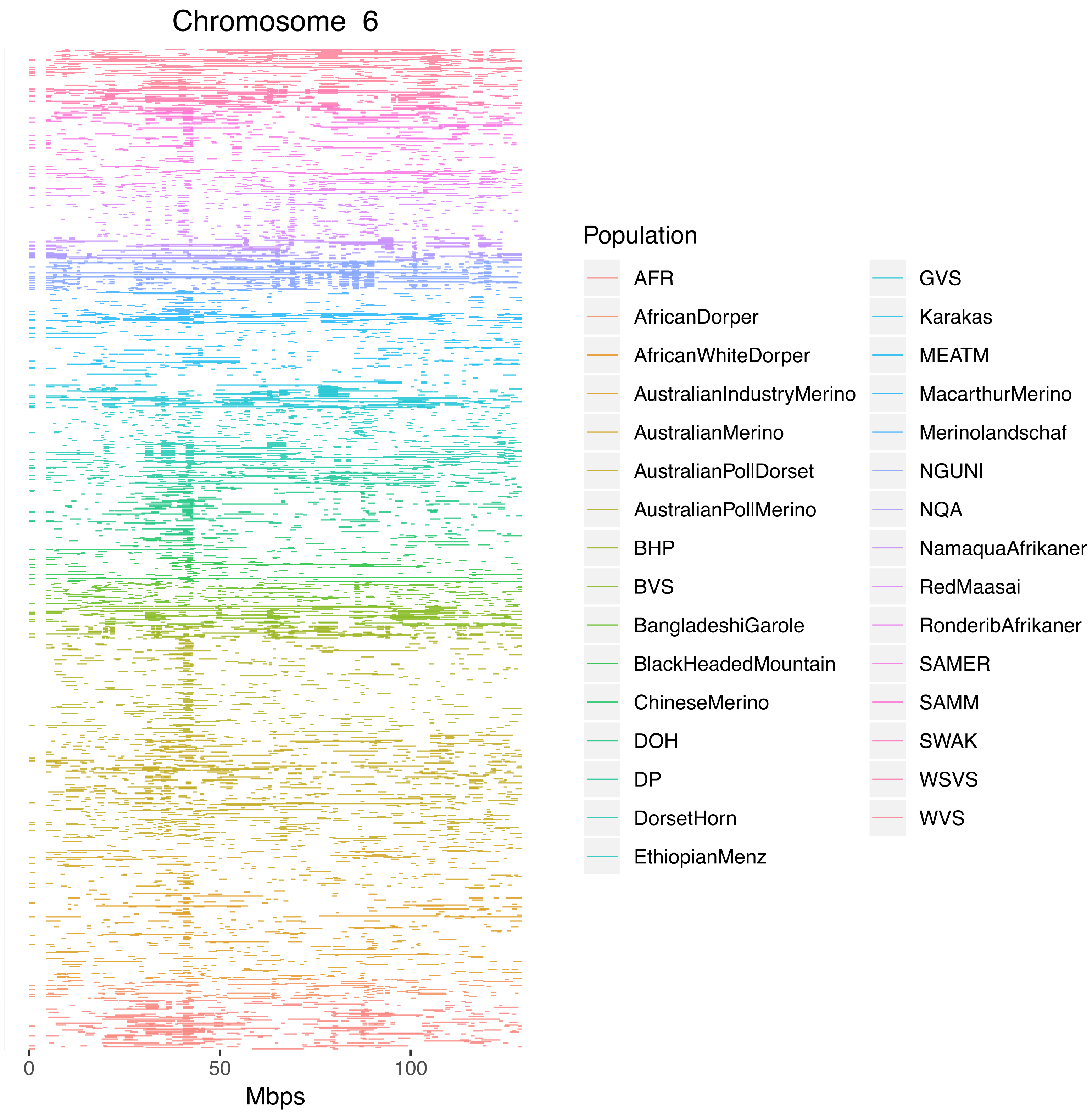

Chromosome 6
0
50
100
Mbps
Population
AFR
AfricanDorper
AfricanWhiteDorper
AustralianIndustryMerino
AustralianMerino
AustralianPollDorset
AustralianPollMerino
BHP
BVS
BangladeshiGarole
BlackHeadedMountain
ChineseMerino
DOH
DP
DorsetHorn
EthiopianMenz
GVS
Karakas
MEATM
MacarthurMerino
Merinolandschaf
NGUNI
NQA
NamaquaAfrikaner
RedMaasai
RonderibAfrikaner
SAMER
SAMM
SWAK
WSVS
WVS

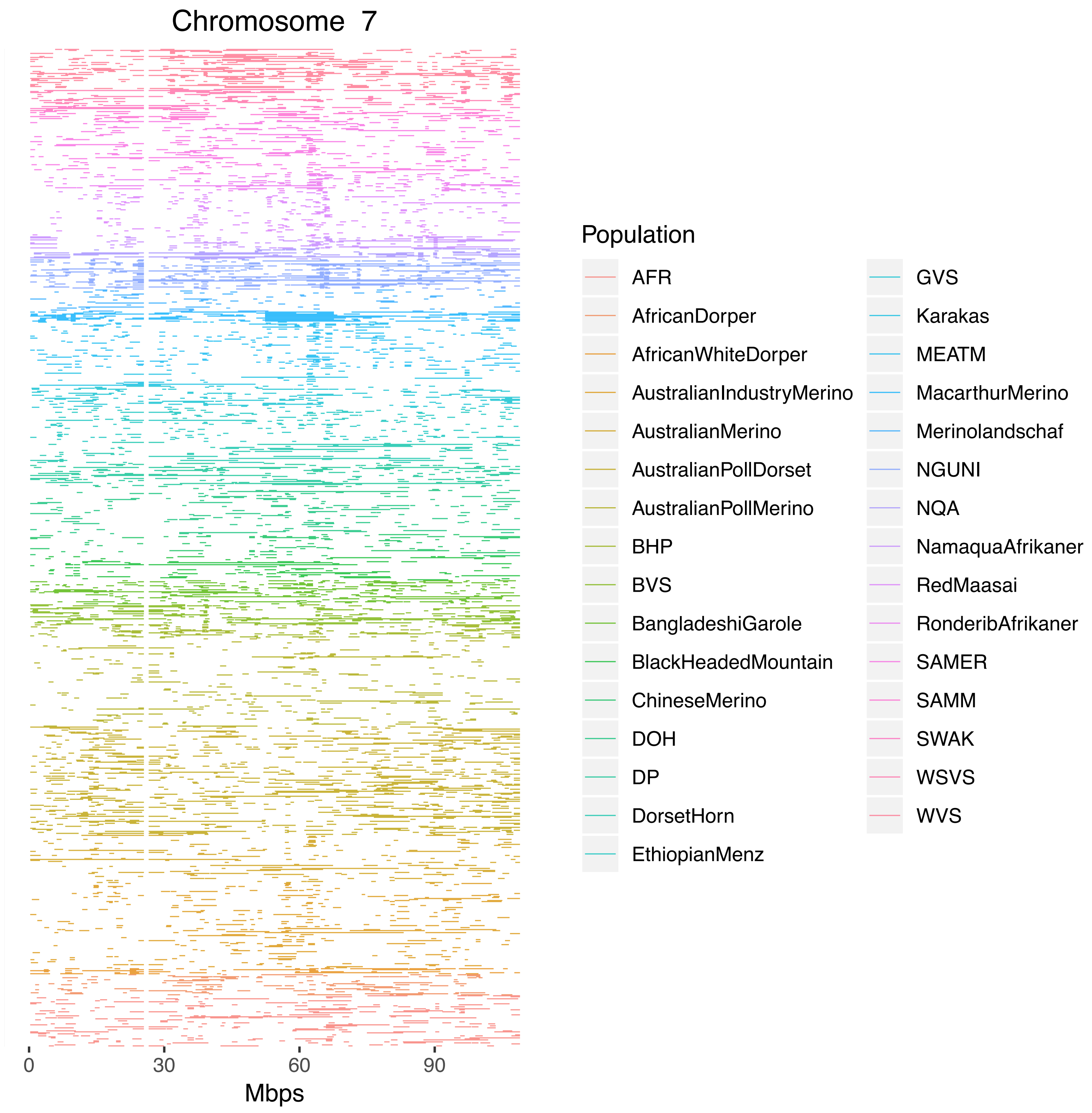
Chromosome 7
0
30
60
90
Mbps
Population
AFR
AfricanDorper
AfricanWhiteDorper
AustralianIndustryMerino
AustralianMerino
AustralianPollDorset
AustralianPollMerino
BHP
BVS
BangladeshiGarole
BlackHeadedMountain
ChineseMerino
DOH
DP
DorsetHorn
EthiopianMenz
GVS
Karakas
MEATM
MacarthurMerino
Merinolandschaf
NGUNI
NQA
NamaquaAfrikaner
RedMaasai
RonderibAfrikaner
SAMER
SAMM
SWAK
WSVS
WVS

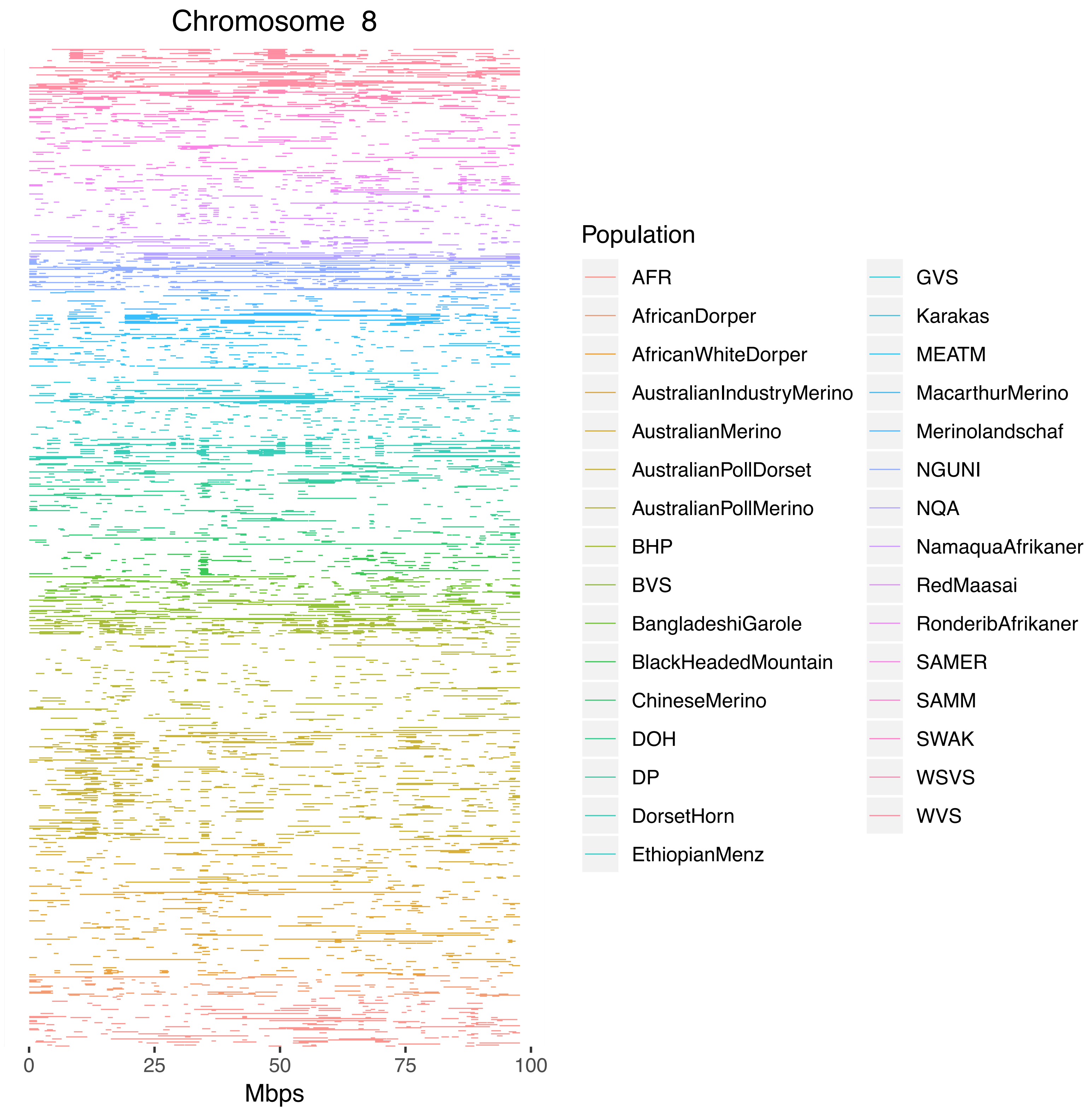

Chromosome 8
0
25
50
75
100
Mbps
Population
AFR
AfricanDorper
AfricanWhiteDorper
AustralianIndustryMerino
AustralianMerino
AustralianPollDorset
AustralianPollMerino
BHP
BVS
BangladeshiGarole
BlackHeadedMountain
ChineseMerino
DOH
DP
DorsetHorn
EthiopianMenz
GVS
Karakas
MEATM
MacarthurMerino
Merinolandschaf
NGUNI
NQA
NamaquaAfrikaner
RedMaasai
RonderibAfrikaner
SAMER
SAMM
SWAK
WSVS
WVS

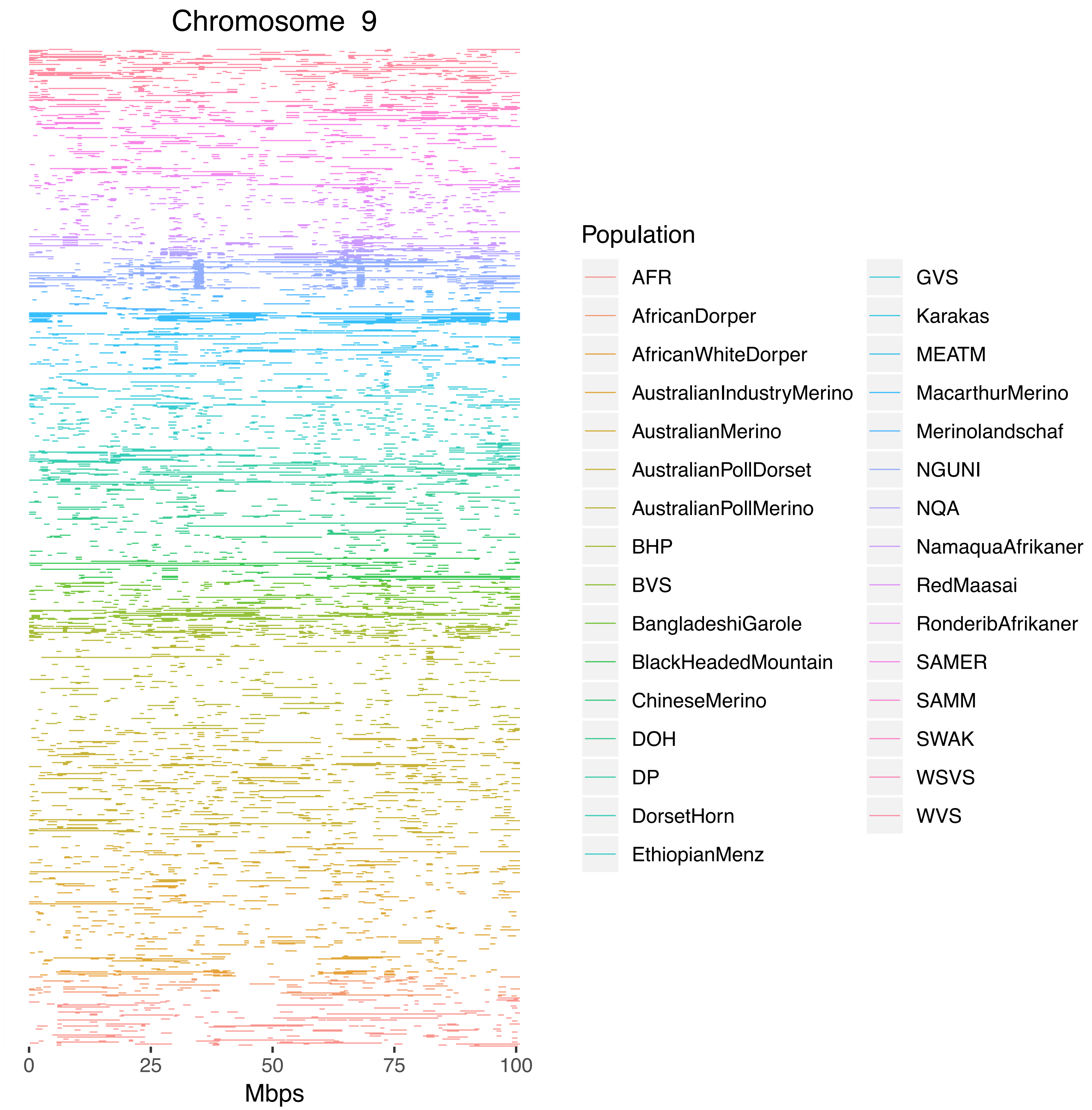

Chromosome 9
0
25
50
75
100
Mbps
Population
AFR
AfricanDorper
AfricanWhiteDorper
AustralianIndustryMerino
AustralianMerino
AustralianPollDorset
AustralianPollMerino
BHP
BVS
BangladeshiGarole
BlackHeadedMountain
ChineseMerino
DOH
DP
DorsetHorn
EthiopianMenz
GVS
Karakas
MEATM
MacarthurMerino
Merinolandschaf
NGUNI
NQA
NamaquaAfrikaner
RedMaasai
RonderibAfrikaner
SAMER
SAMM
SWAK
WSVS
WVS

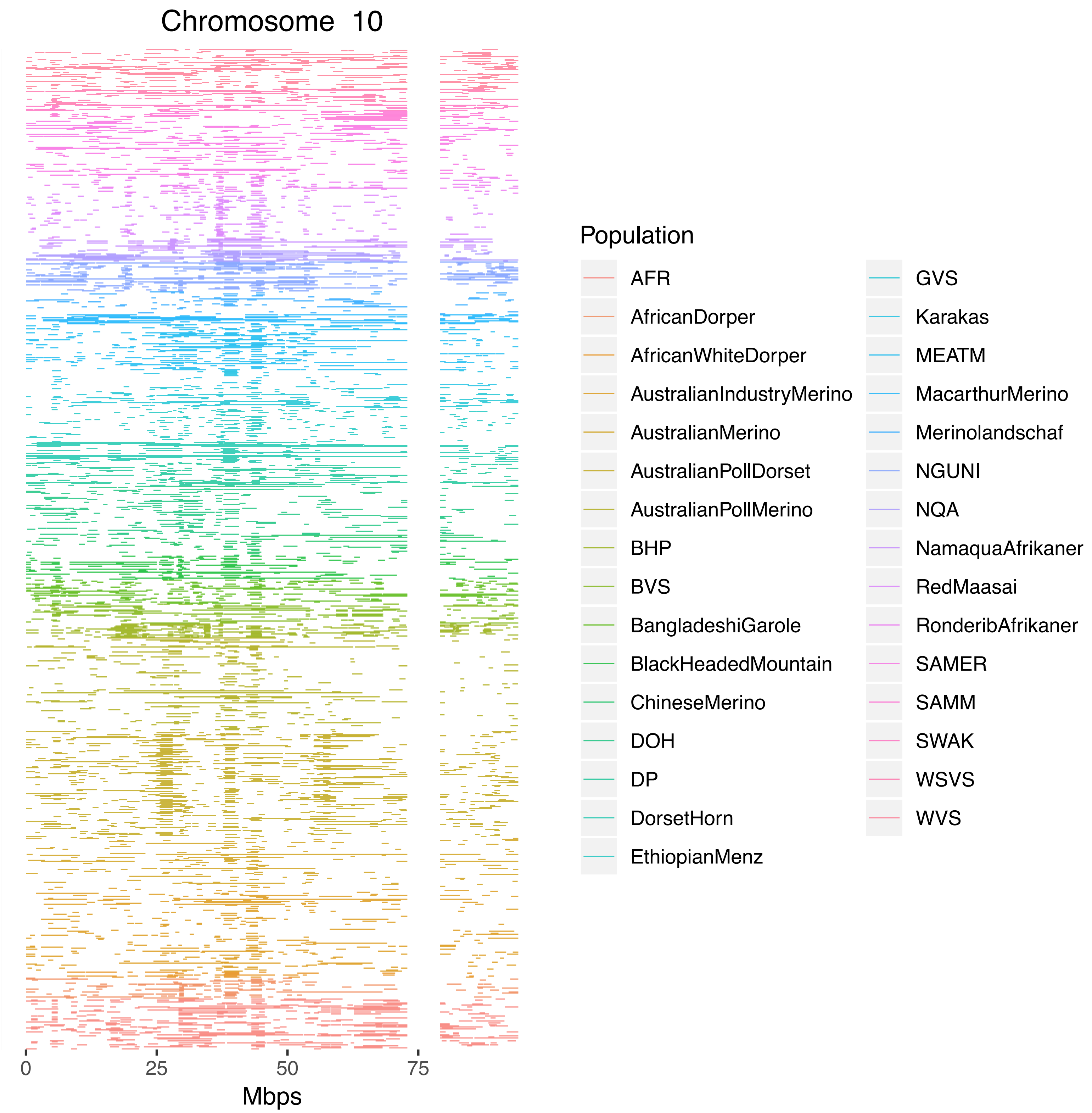
Chromosome 10
0
25
50
75
Mbps
Population
AFR
AfricanDorper
AfricanWhiteDorper
AustralianIndustryMerino
AustralianMerino
AustralianPollDorset
AustralianPollMerino
BHP
BVS
BangladeshiGarole
BlackHeadedMountain
ChineseMerino
DOH
DP
DorsetHorn
EthiopianMenz
GVS
Karakas
MEATM
MacarthurMerino
Merinolandschaf
NGUNI
NQA
NamaquaAfrikaner
RedMaasai
RonderibAfrikaner
SAMER
SAMM
SWAK
WSVS
WVS

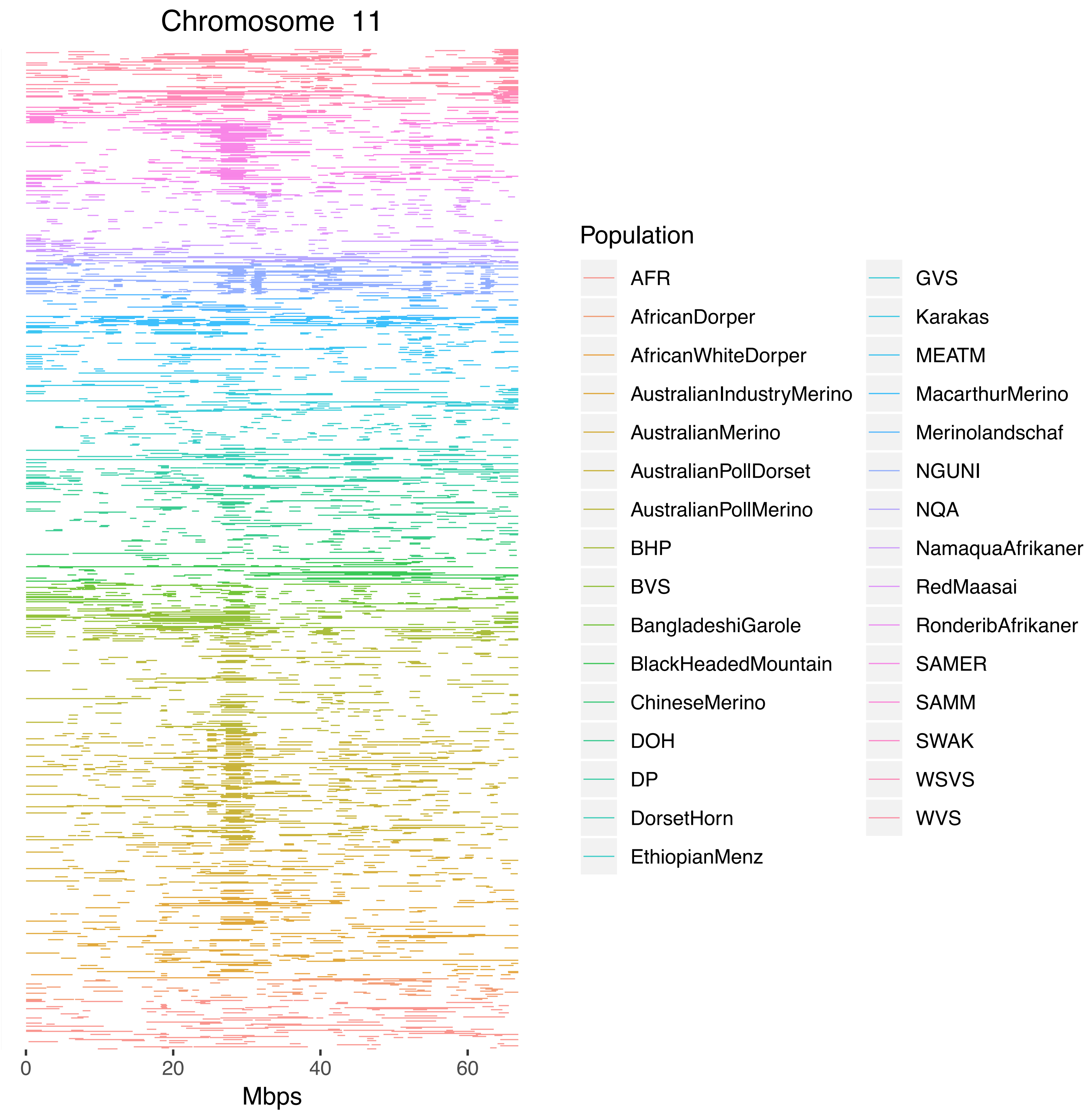

Chromosome 11
0
20
40
60
Mbps
Population
AFR
AfricanDorper
AfricanWhiteDorper
AustralianIndustryMerino
AustralianMerino
AustralianPollDorset
AustralianPollMerino
BHP
BVS
BangladeshiGarole
BlackHeadedMountain
ChineseMerino
DOH
DP
DorsetHorn
EthiopianMenz
GVS
Karakas
MEATM
MacarthurMerino
Merinolandschaf
NGUNI
NQA
NamaquaAfrikaner
RedMaasai
RonderibAfrikaner
SAMER
SAMM
SWAK
WSVS
WVS

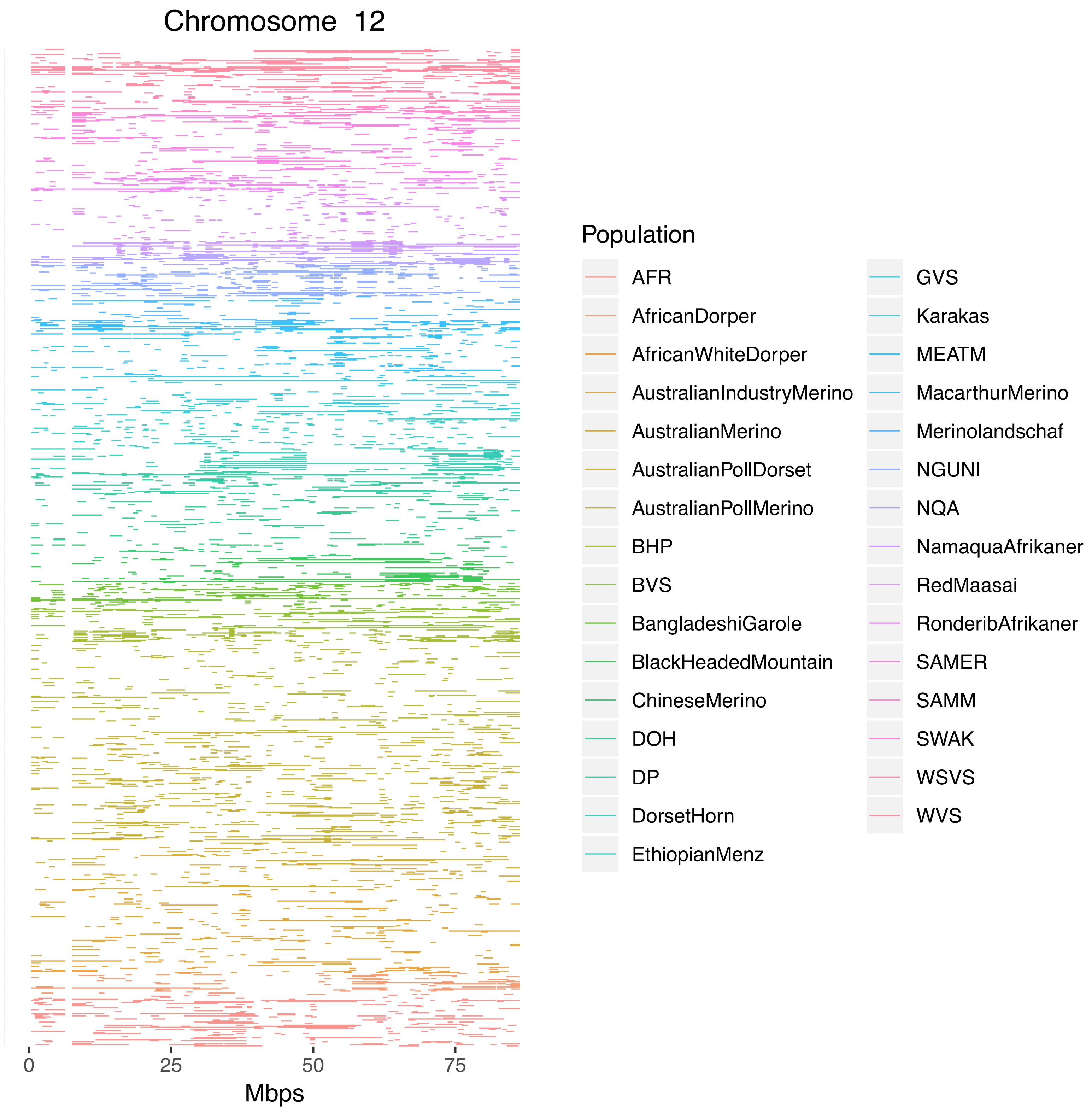

Chromosome 12
0
25
50
75
Mbps
Population
AFR
AfricanDorper
AfricanWhiteDorper
AustralianIndustryMerino
AustralianMerino
AustralianPollDorset
AustralianPollMerino
BHP
BVS
BangladeshiGarole
BlackHeadedMountain
ChineseMerino
DOH
DP
DorsetHorn
EthiopianMenz
GVS
Karakas
MEATM
MacarthurMerino
Merinolandschaf
NGUNI
NQA
NamaquaAfrikaner
RedMaasai
RonderibAfrikaner
SAMER
SAMM
SWAK
WSVS
WVS

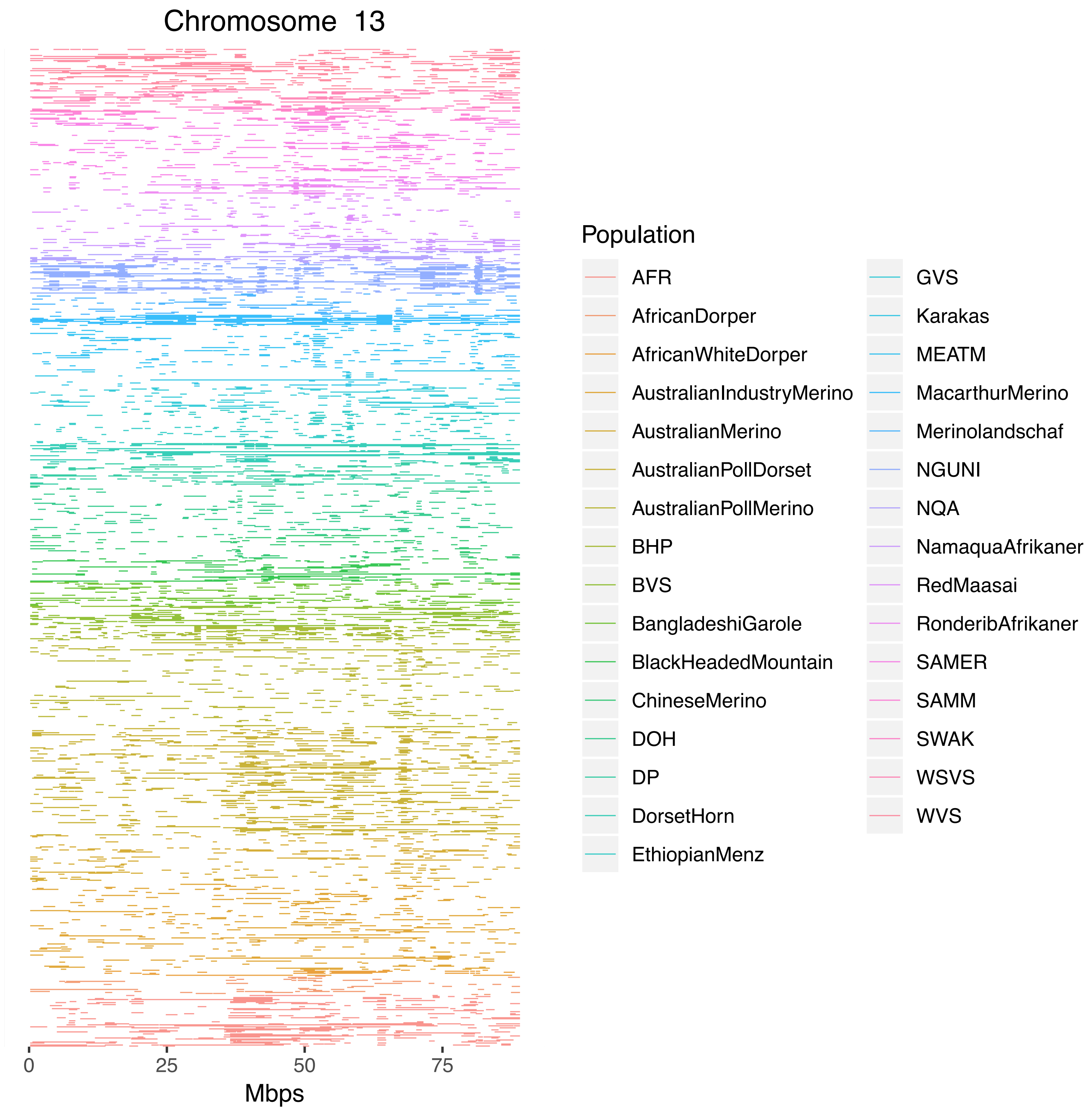
Chromosome 13
0
25
50
75
Mbps
Population
AFR
AfricanDorper
AfricanWhiteDorper
AustralianIndustryMerino
AustralianMerino
AustralianPollDorset
AustralianPollMerino
BHP
BVS
BangladeshiGarole
BlackHeadedMountain
ChineseMerino
DOH
DP
DorsetHorn
EthiopianMenz
GVS
Karakas
MEATM
MacarthurMerino
Merinolandschaf
NGUNI
NQA
NamaquaAfrikaner
RedMaasai
RonderibAfrikaner
SAMER
SAMM
SWAK
WSVS
WVS

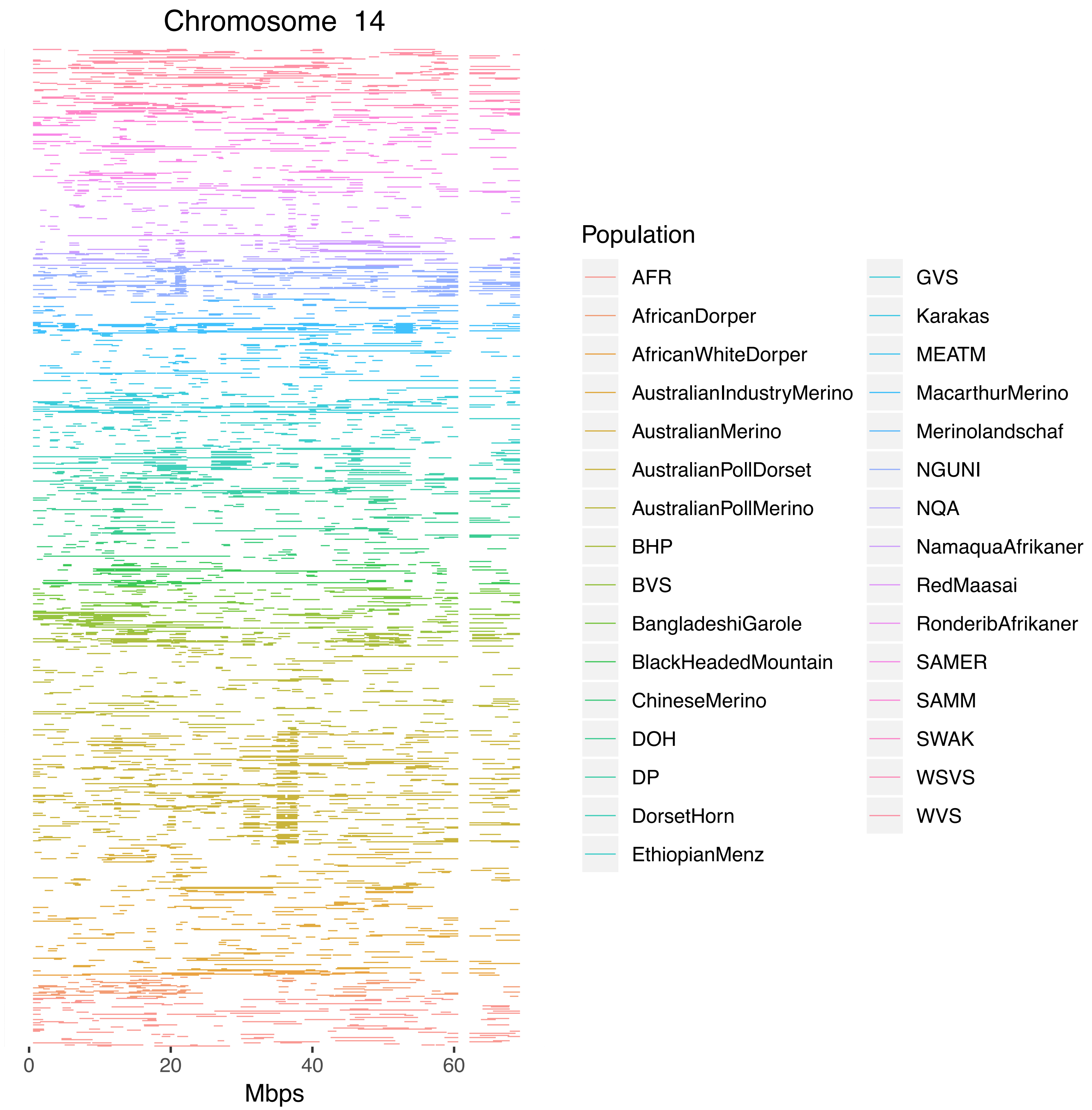

Chromosome 14
0
20
40
60
Mbps
Population
AFR
AfricanDorper
AfricanWhiteDorper
AustralianIndustryMerino
AustralianMerino
AustralianPollDorset
AustralianPollMerino
BHP
BVS
BangladeshiGarole
BlackHeadedMountain
ChineseMerino
DOH
DP
DorsetHorn
EthiopianMenz
GVS
Karakas
MEATM
MacarthurMerino
Merinolandschaf
NGUNI
NQA
NamaquaAfrikaner
RedMaasai
RonderibAfrikaner
SAMER
SAMM
SWAK
WSVS
WVS

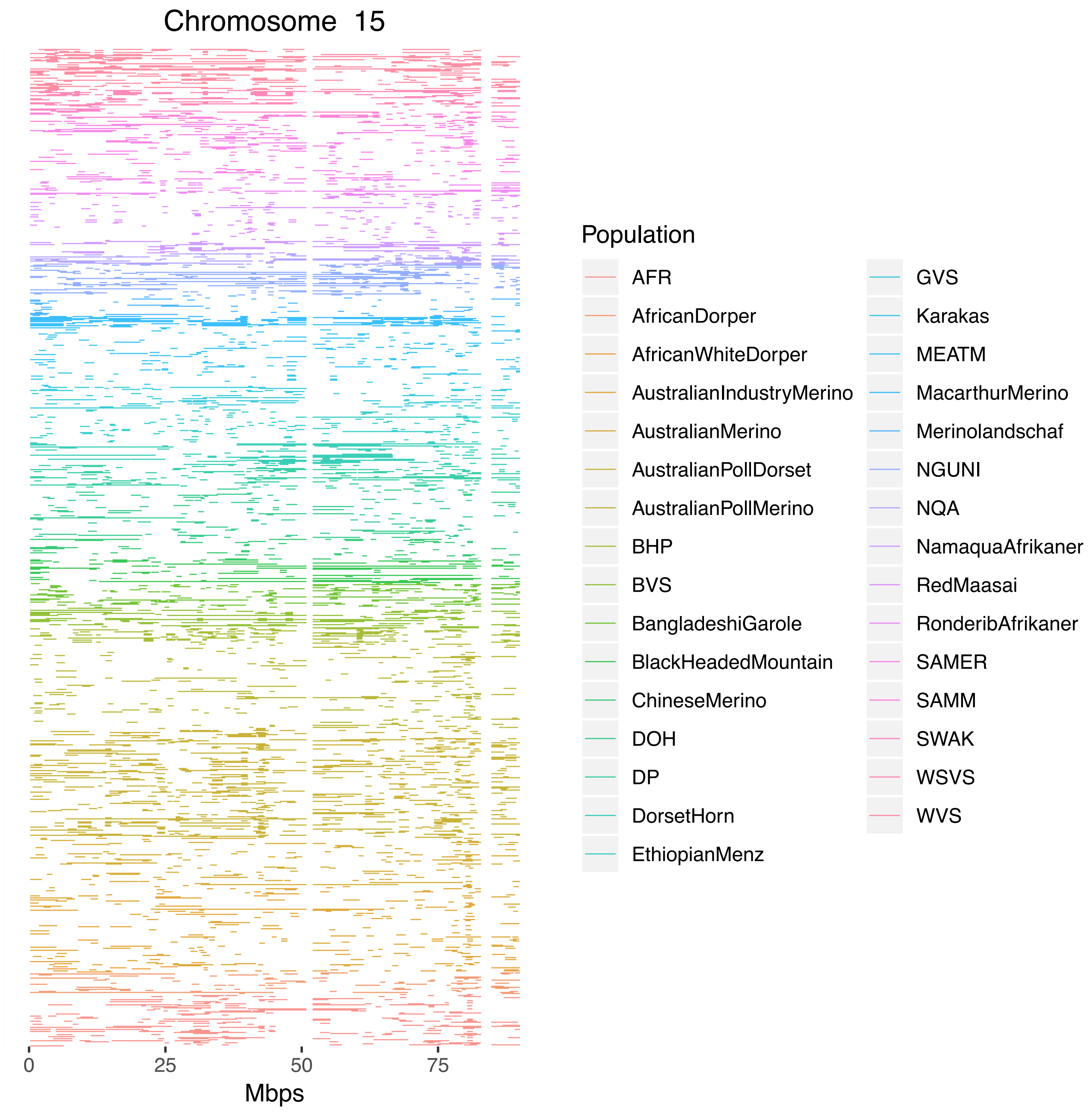
Chromosome 15
0
25
50
75
Mbps
Population
AFR
AfricanDorper
AfricanWhiteDorper
AustralianIndustryMerino
AustralianMerino
AustralianPollDorset
AustralianPollMerino
BHP
BVS
BangladeshiGarole
BlackHeadedMountain
ChineseMerino
DOH
DP
DorsetHorn
EthiopianMenz
GVS
Karakas
MEATM
MacarthurMerino
Merinolandschaf
NGUNI
NQA
NamaquaAfrikaner
RedMaasai
RonderibAfrikaner
SAMER
SAMM
SWAK
WSVS
WVS

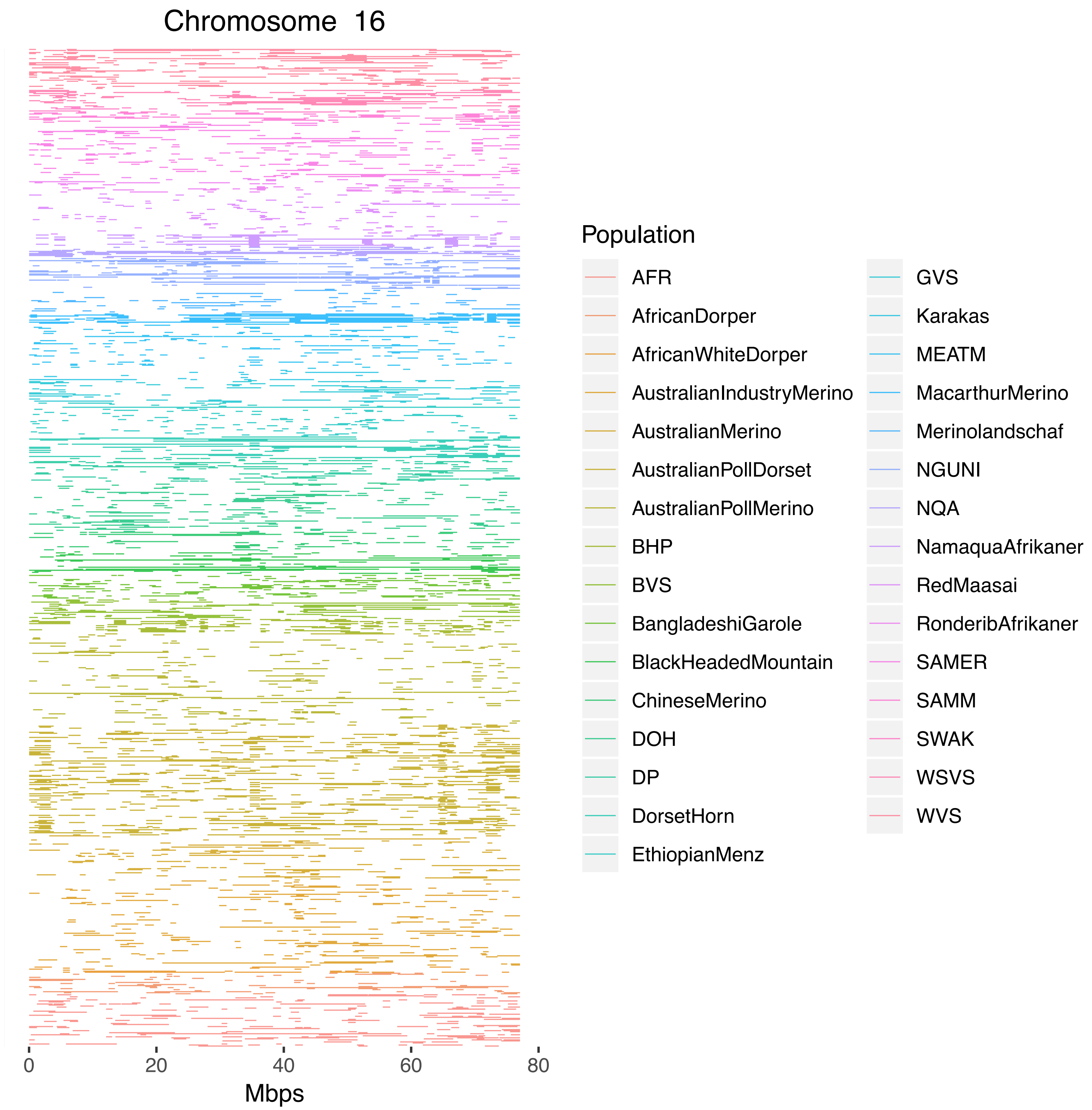

Chromosome 16
0
20
40
60
80
Mbps
Population
AFR
AfricanDorper
AfricanWhiteDorper
AustralianIndustryMerino
AustralianMerino
AustralianPollDorset
AustralianPollMerino
BHP
BVS
BangladeshiGarole
BlackHeadedMountain
ChineseMerino
DOH
DP
DorsetHorn
EthiopianMenz
GVS
Karakas
MEATM
MacarthurMerino
Merinolandschaf
NGUNI
NQA
NamaquaAfrikaner
RedMaasai
RonderibAfrikaner
SAMER
SAMM
SWAK
WSVS
WVS

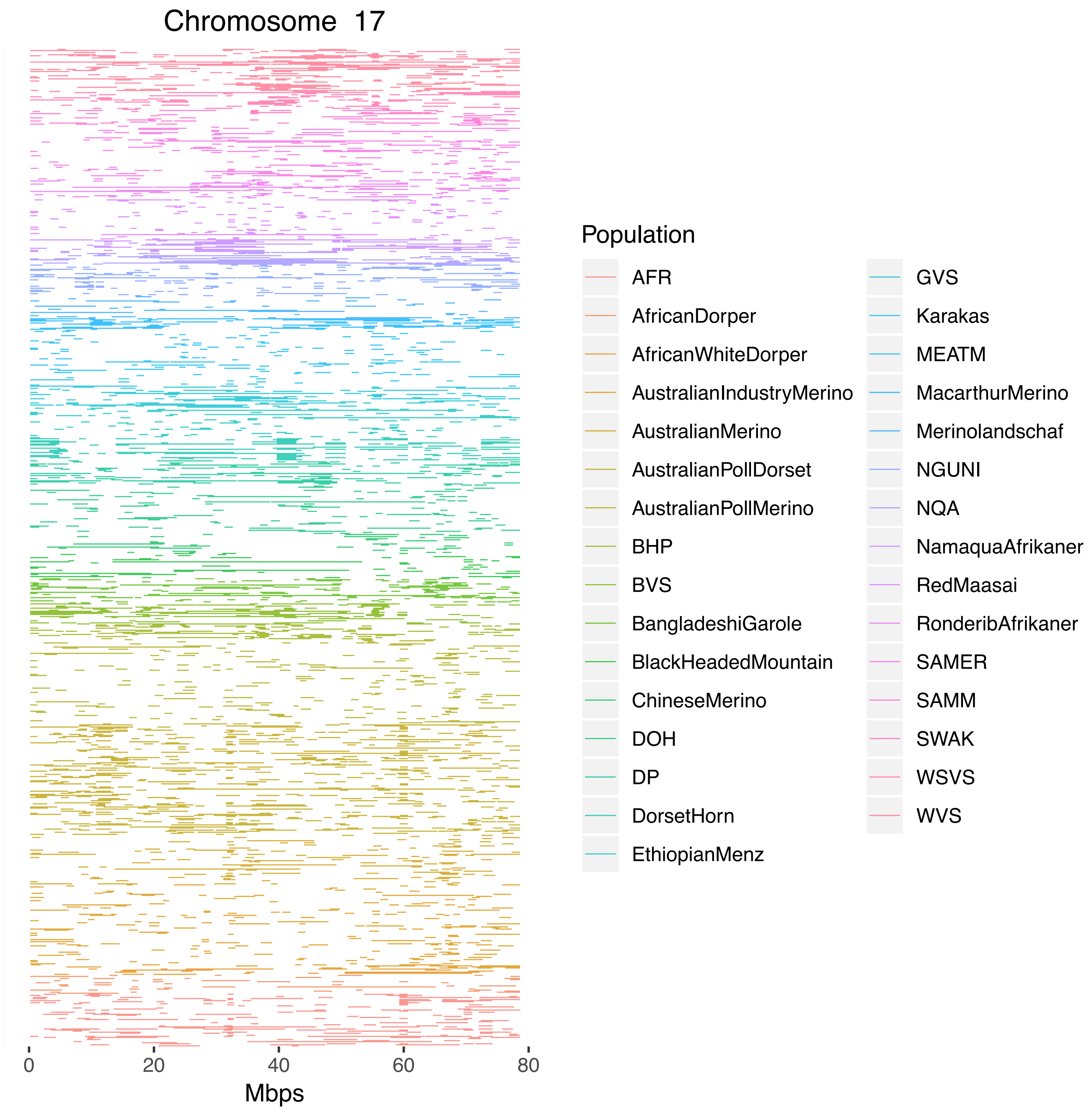
Chromosome 17
0
20
40
60
80
Mbps
Population
AFR
AfricanDorper
AfricanWhiteDorper
AustralianIndustryMerino
AustralianMerino
AustralianPollDorset
AustralianPollMerino
BHP
BVS
BangladeshiGarole
BlackHeadedMountain
ChineseMerino
DOH
DP
DorsetHorn
EthiopianMenz
GVS
Karakas
MEATM
MacarthurMerino
Merinolandschaf
NGUNI
NQA
NamaquaAfrikaner
RedMaasai
RonderibAfrikaner
SAMER
SAMM
SWAK
WSVS
WVS

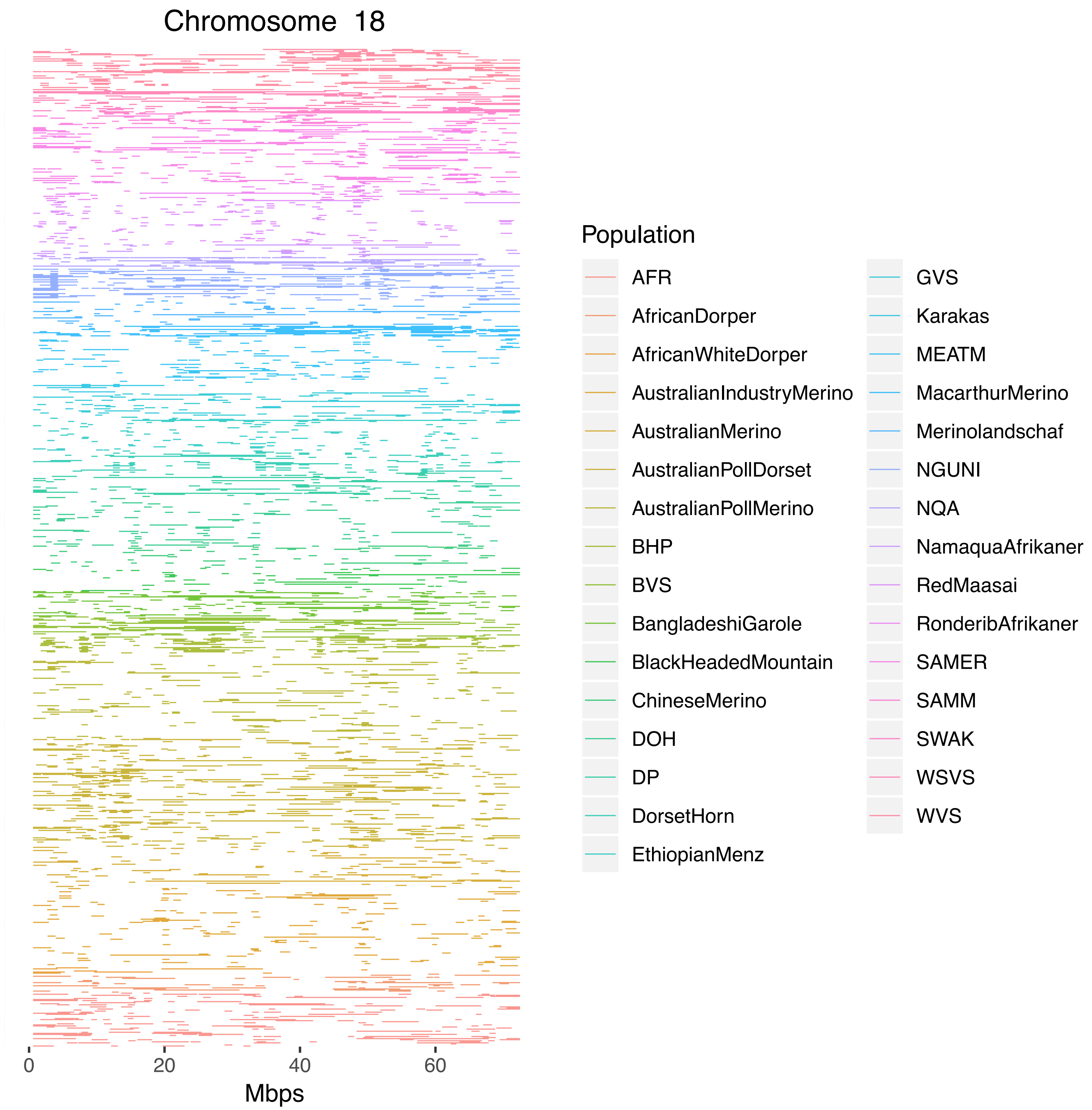

Chromosome 18
0
20
40
60
Mbps
Population
AFR
AfricanDorper
AfricanWhiteDorper
AustralianIndustryMerino
AustralianMerino
AustralianPollDorset
AustralianPollMerino
BHP
BVS
BangladeshiGarole
BlackHeadedMountain
ChineseMerino
DOH
DP
DorsetHorn
EthiopianMenz
GVS
Karakas
MEATM
MacarthurMerino
Merinolandschaf
NGUNI
NQA
NamaquaAfrikaner
RedMaasai
RonderibAfrikaner
SAMER
SAMM
SWAK
WSVS
WVS

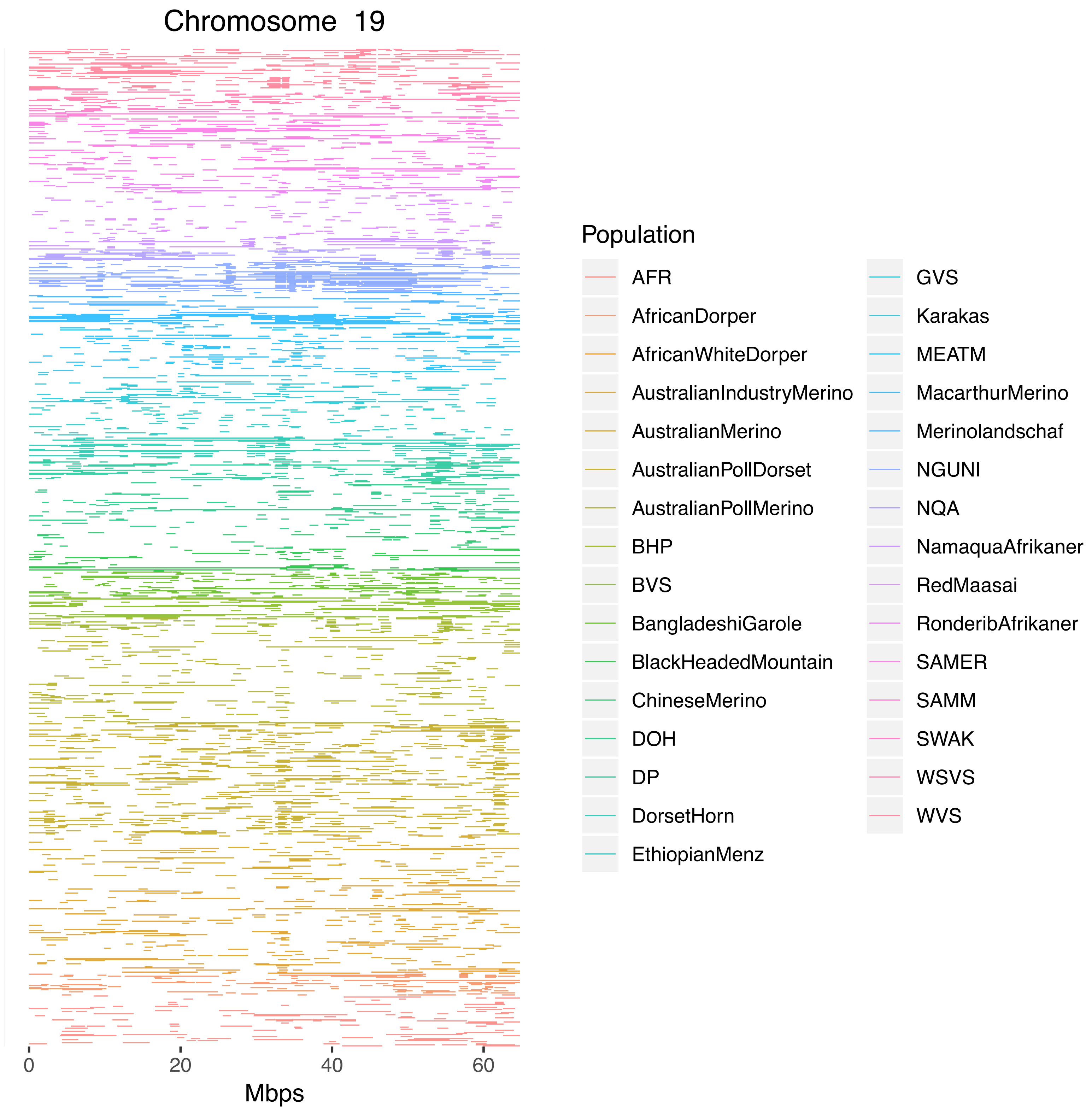
Chromosome 19
0
20
40
60
Mbps
Population
AFR
AfricanDorper
AfricanWhiteDorper
AustralianIndustryMerino
AustralianMerino
AustralianPollDorset
AustralianPollMerino
BHP
BVS
BangladeshiGarole
BlackHeadedMountain
ChineseMerino
DOH
DP
DorsetHorn
EthiopianMenz
GVS
Karakas
MEATM
MacarthurMerino
Merinolandschaf
NGUNI
NQA
NamaquaAfrikaner
RedMaasai
RonderibAfrikaner
SAMER
SAMM
SWAK
WSVS
WVS

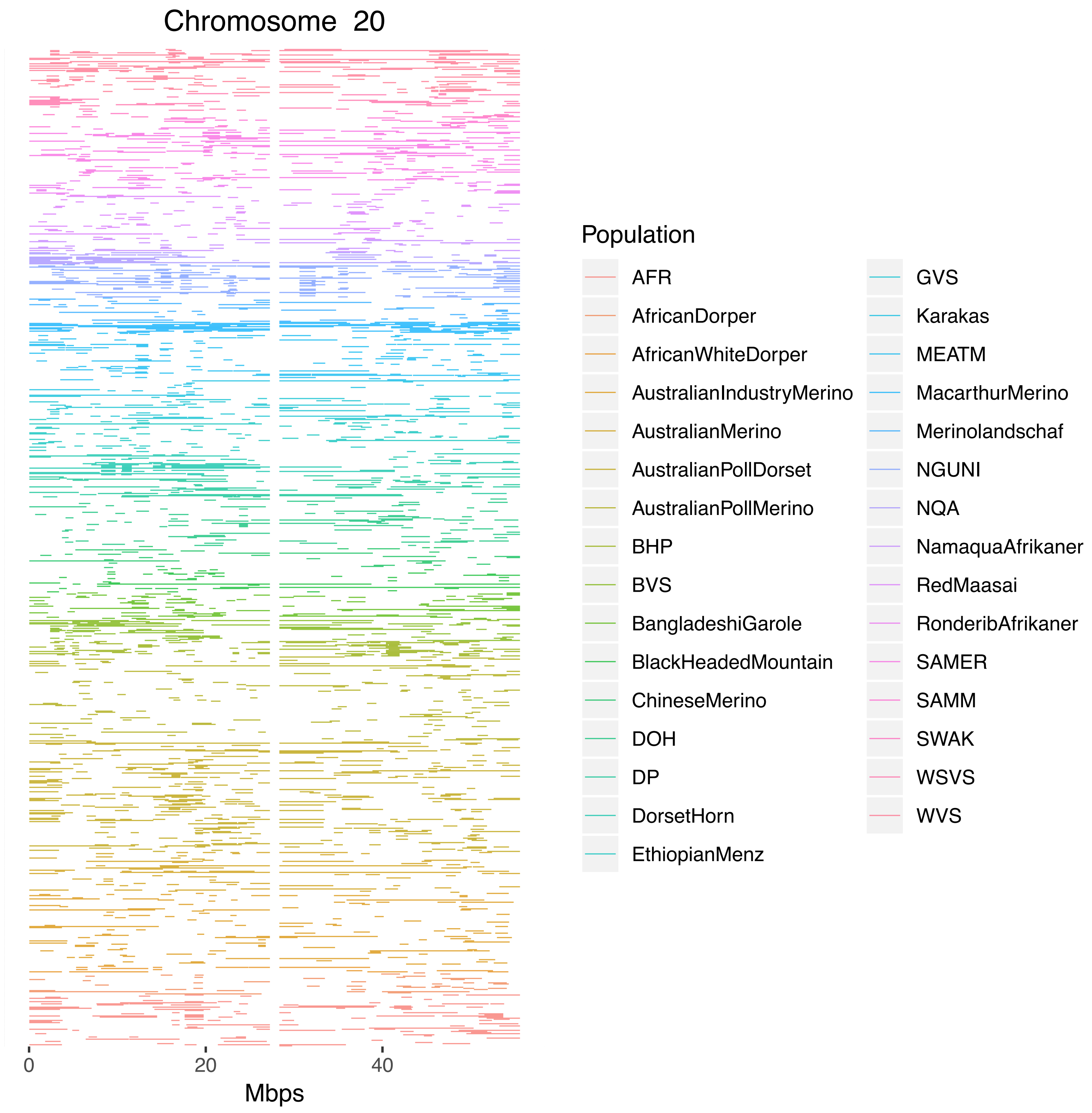

Chromosome 20
0
20
40
Mbps
Population
AFR
AfricanDorper
AfricanWhiteDorper
AustralianIndustryMerino
AustralianMerino
AustralianPollDorset
AustralianPollMerino
BHP
BVS
BangladeshiGarole
BlackHeadedMountain
ChineseMerino
DOH
DP
DorsetHorn
EthiopianMenz
GVS
Karakas
MEATM
MacarthurMerino
Merinolandschaf
NGUNI
NQA
NamaquaAfrikaner
RedMaasai
RonderibAfrikaner
SAMER
SAMM
SWAK
WSVS
WVS

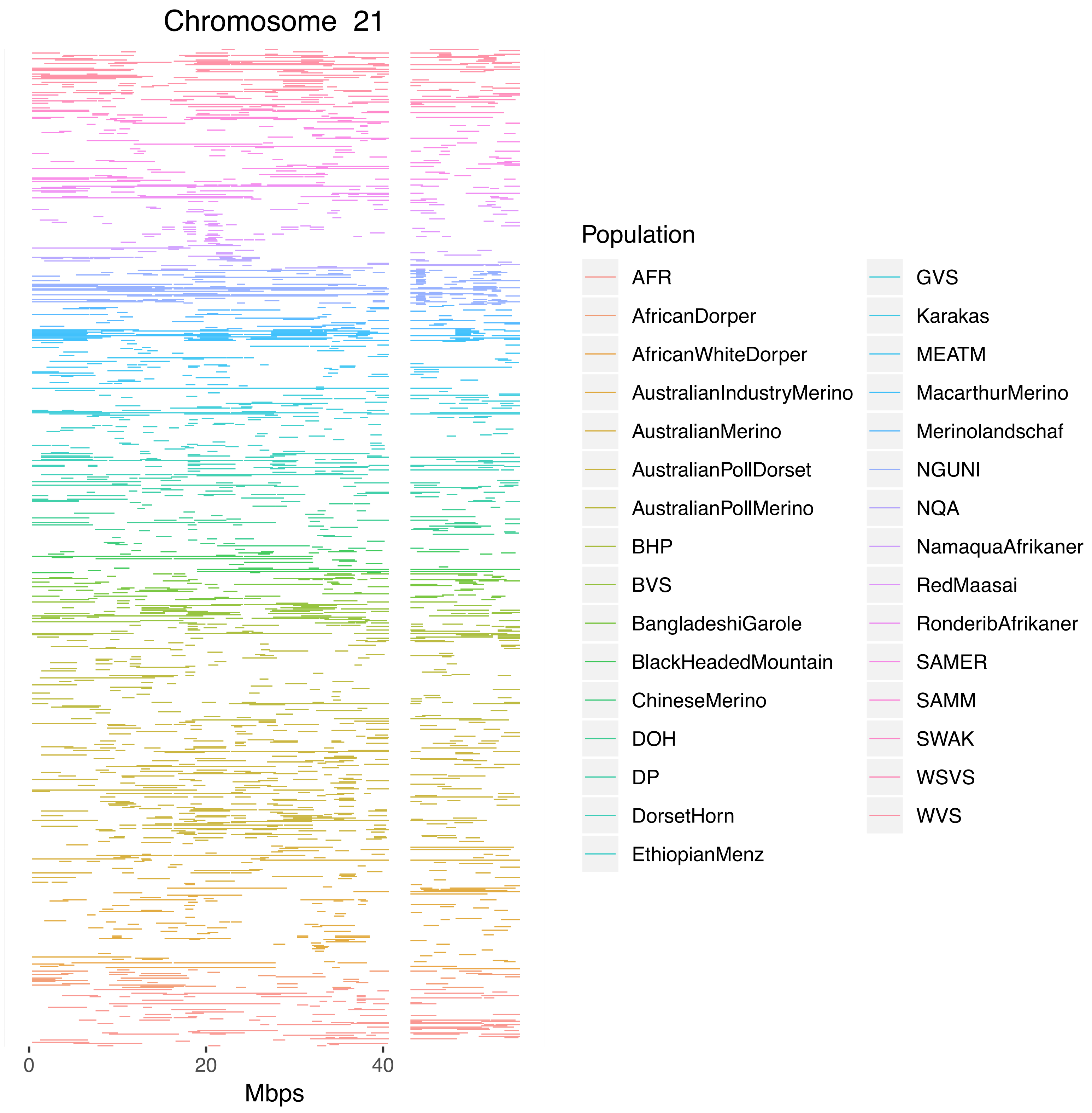

Chromosome 21
0
20
40
Mbps
Population
AFR
AfricanDorper
AfricanWhiteDorper
AustralianIndustryMerino
AustralianMerino
AustralianPollDorset
AustralianPollMerino
BHP
BVS
BangladeshiGarole
BlackHeadedMountain
ChineseMerino
DOH
DP
DorsetHorn
EthiopianMenz
GVS
Karakas
MEATM
MacarthurMerino
Merinolandschaf
NGUNI
NQA
NamaquaAfrikaner
RedMaasai
RonderibAfrikaner
SAMER
SAMM
SWAK
WSVS
WVS

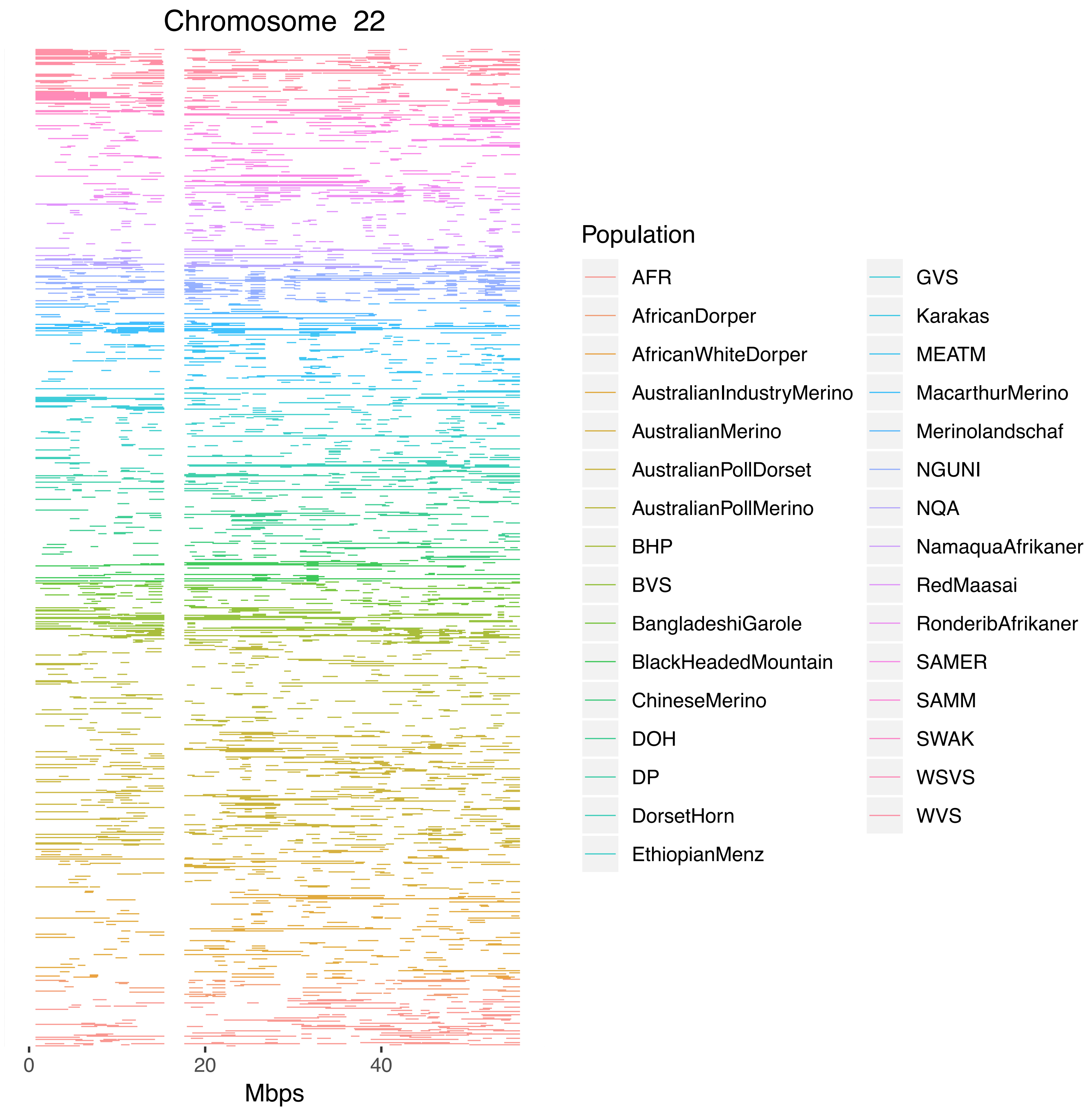
Chromosome 22
0
20
40
Mbps
Population
AFR
AfricanDorper
AfricanWhiteDorper
AustralianIndustryMerino
AustralianMerino
AustralianPollDorset
AustralianPollMerino
BHP
BVS
BangladeshiGarole
BlackHeadedMountain
ChineseMerino
DOH
DP
DorsetHorn
EthiopianMenz
GVS
Karakas
MEATM
MacarthurMerino
Merinolandschaf
NGUNI
NQA
NamaquaAfrikaner
RedMaasai
RonderibAfrikaner
SAMER
SAMM
SWAK
WSVS
WVS

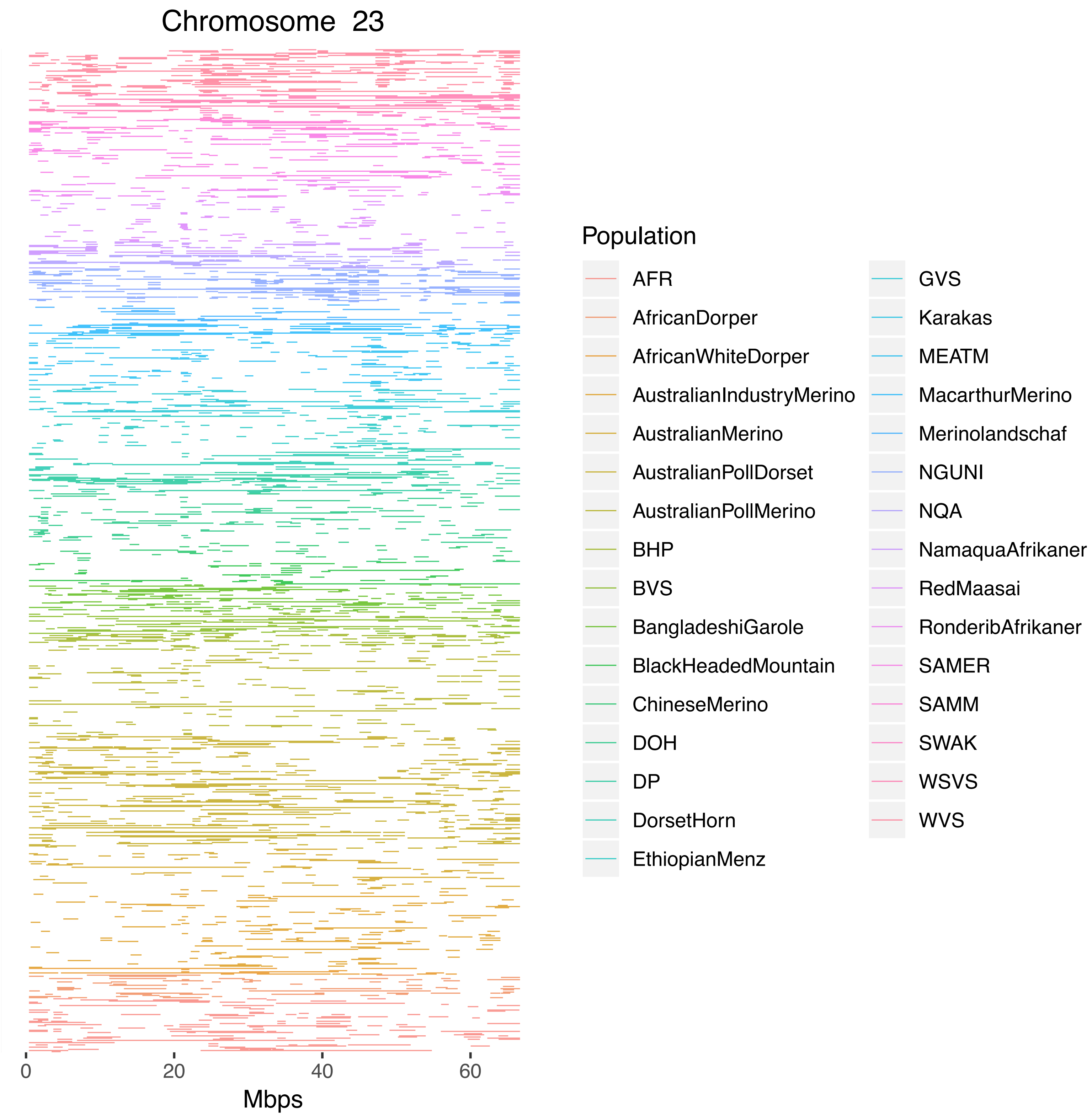

Chromosome 23
0
20
40
60
Mbps
Population
AFR
AfricanDorper
AfricanWhiteDorper
AustralianIndustryMerino
AustralianMerino
AustralianPollDorset
AustralianPollMerino
BHP
BVS
BangladeshiGarole
BlackHeadedMountain
ChineseMerino
DOH
DP
DorsetHorn
EthiopianMenz
GVS
Karakas
MEATM
MacarthurMerino
Merinolandschaf
NGUNI
NQA
NamaquaAfrikaner
RedMaasai
RonderibAfrikaner
SAMER
SAMM
SWAK
WSVS
WVS

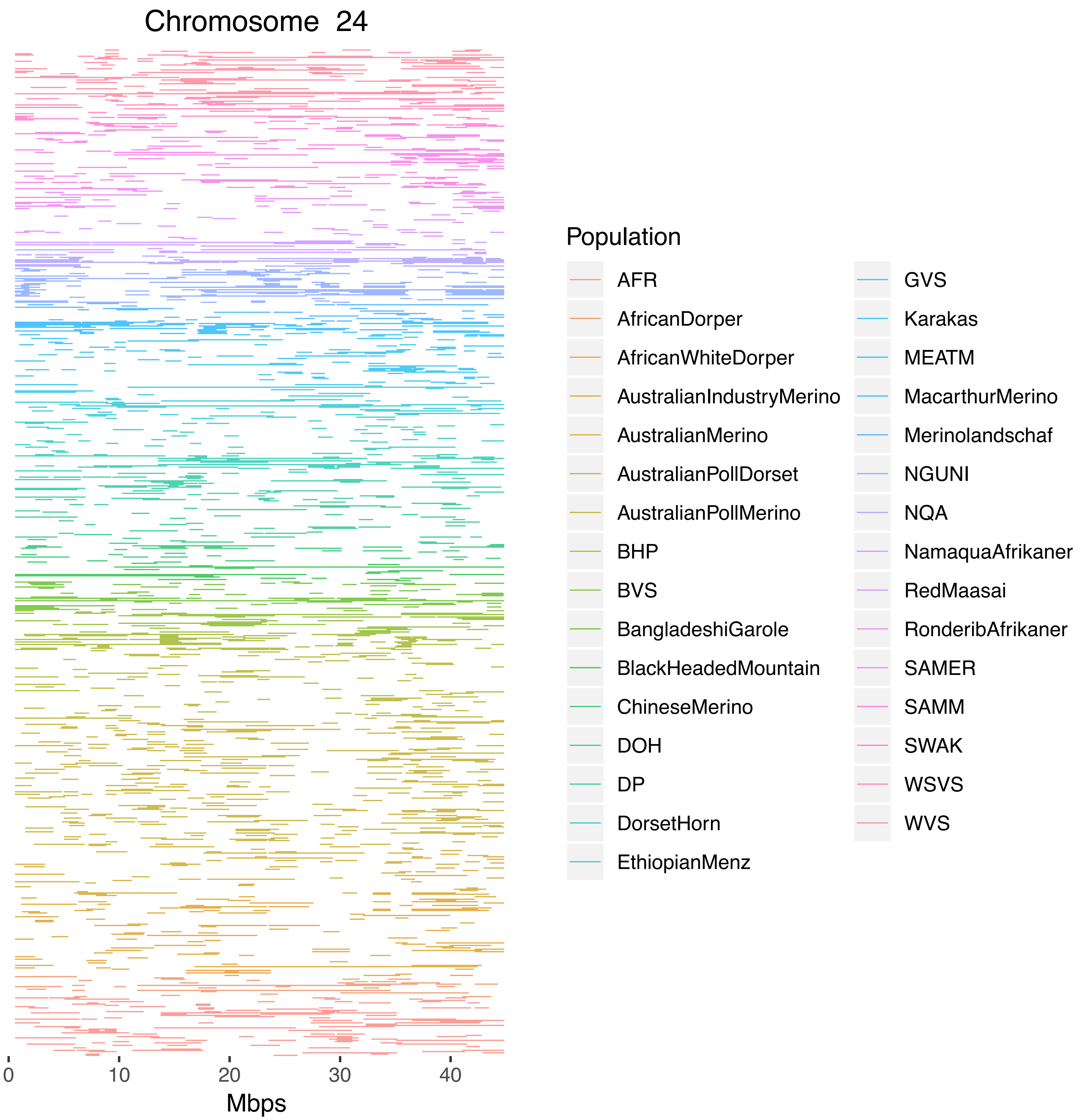

Chromosome 24
0
10
20
30
40
Mbps
Population
AFR
AfricanDorper
AfricanWhiteDorper
AustralianIndustryMerino
AustralianMerino
AustralianPollDorset
AustralianPollMerino
BHP
BVS
BangladeshiGarole
BlackHeadedMountain
ChineseMerino
DOH
DP
DorsetHorn
EthiopianMenz
GVS
Karakas
MEATM
MacarthurMerino
Merinolandschaf
NGUNI
NQA
NamaquaAfrikaner
RedMaasai
RonderibAfrikaner
SAMER
SAMM
SWAK
WSVS
WVS

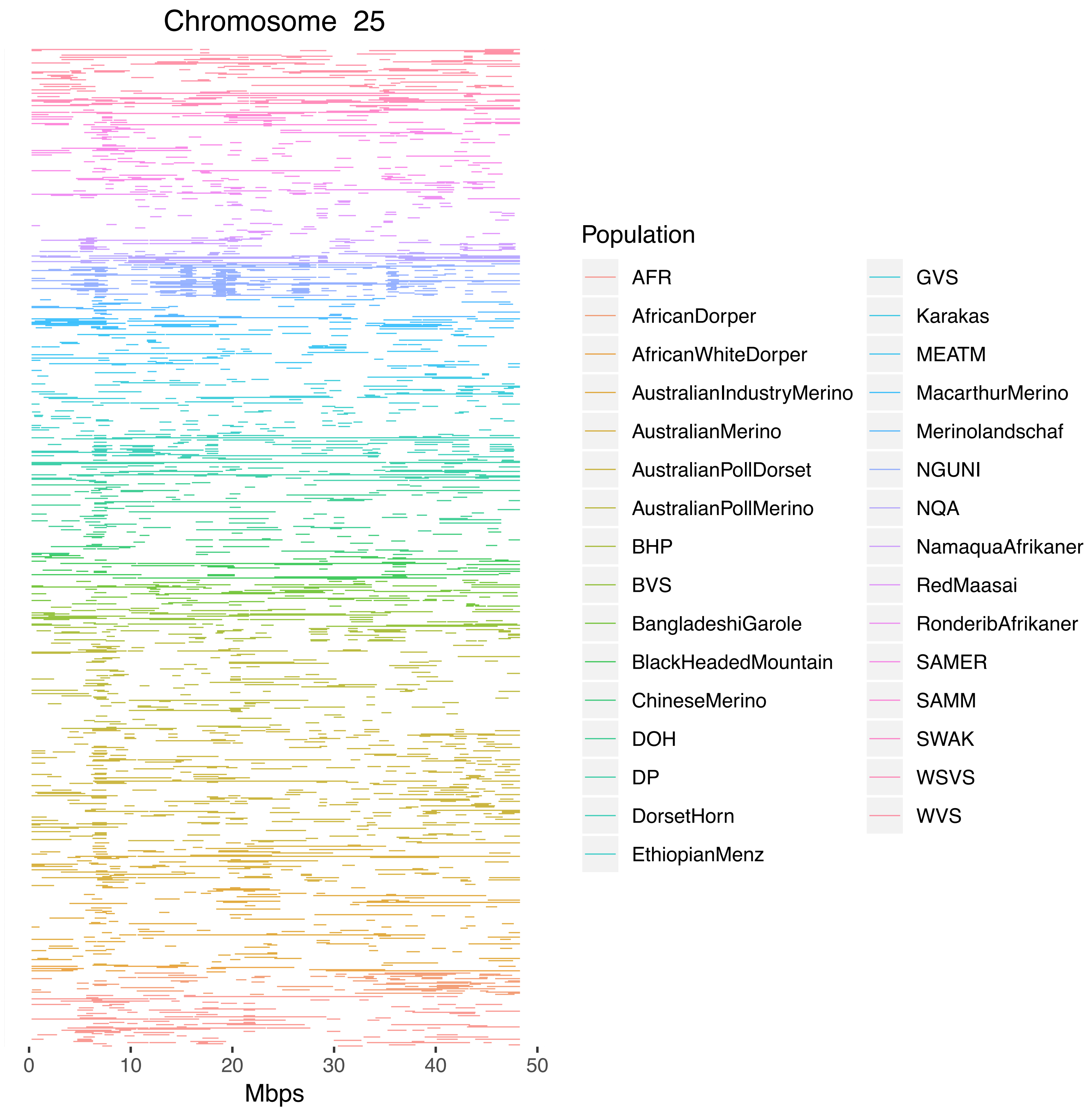

Chromosome 25
0
10
20
30
40
50
Mbps
Population
AFR
AfricanDorper
AfricanWhiteDorper
AustralianIndustryMerino
AustralianMerino
AustralianPollDorset
AustralianPollMerino
BHP
BVS
BangladeshiGarole
BlackHeadedMountain
ChineseMerino
DOH
DP
DorsetHorn
EthiopianMenz
GVS
Karakas
MEATM
MacarthurMerino
Merinolandschaf
NGUNI
NQA
NamaquaAfrikaner
RedMaasai
RonderibAfrikaner
SAMER
SAMM
SWAK
WSVS
WVS

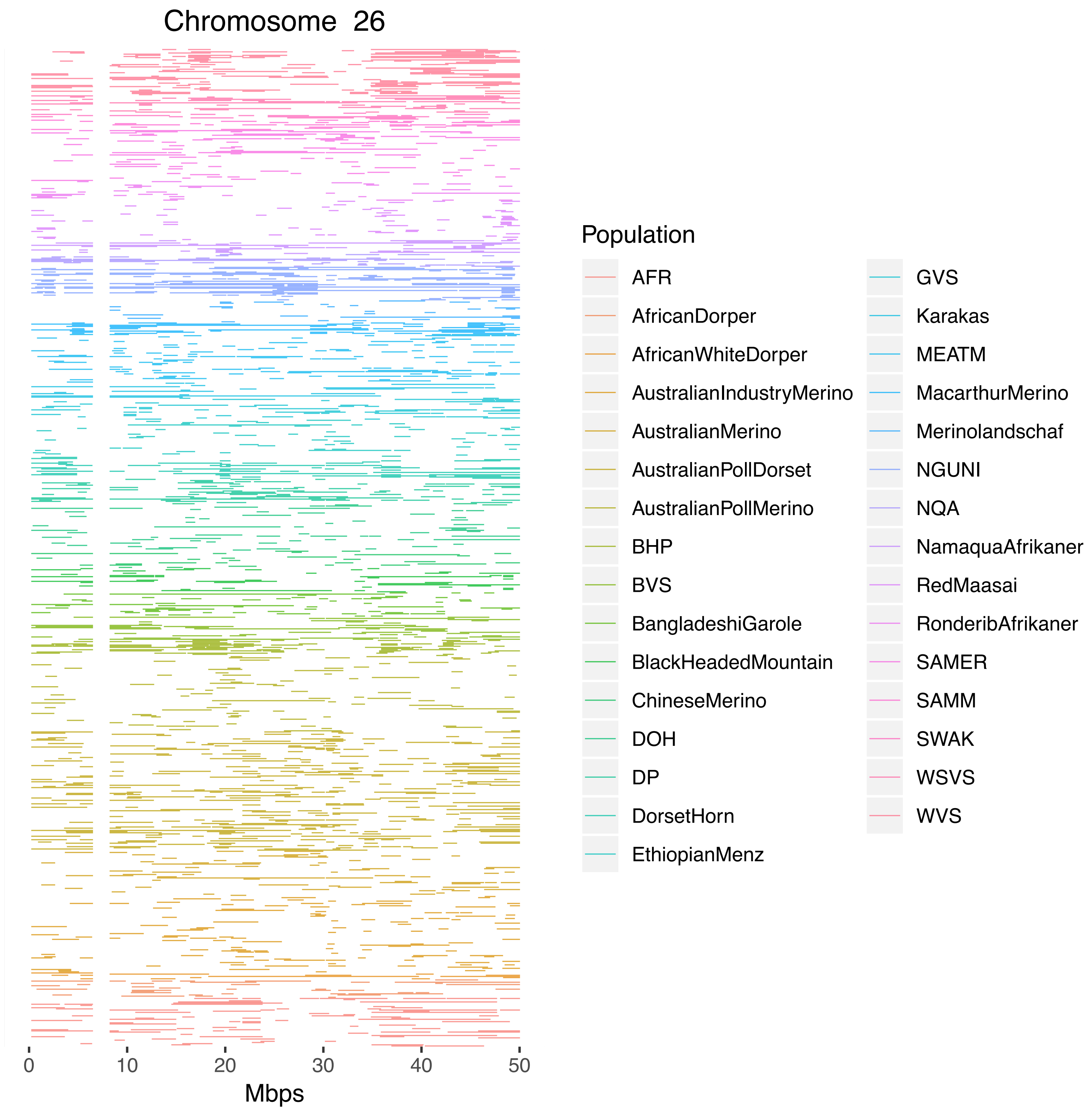
Chromosome 26
0
10
20
30
40
50
Mbps
Population
AFR
AfricanDorper
AfricanWhiteDorper
AustralianIndustryMerino
AustralianMerino
AustralianPollDorset
AustralianPollMerino
BHP
BVS
BangladeshiGarole
BlackHeadedMountain
ChineseMerino
DOH
DP
DorsetHorn
EthiopianMenz
GVS
Karakas
MEATM
MacarthurMerino
Merinolandschaf
NGUNI
NQA
NamaquaAfrikaner
RedMaasai
RonderibAfrikaner
SAMER
SAMM
SWAK
WSVS
WVS
